# Melatonin induces the rejuvenation of long-term ex vivo expanded periodontal ligament stem cells by modulating the autophagic process

**DOI:** 10.1186/s13287-021-02322-9

**Published:** 2021-04-29

**Authors:** Yi-Zhou Tan, Xin-Yue Xu, Ji-Min Dai, Yuan Yin, Xiao-Tao He, Yi-Lin Zhang, Tian-Xiao Zhu, Ying An, Bei-Min Tian, Fa-Ming Chen

**Affiliations:** 1 Department of Periodontology, School of Stomatology, State Key Laboratory of Military Stomatology, National Clinical Research Center for Oral Diseases and Shaanxi Engineering Research Center for Dental Materials and Advanced Manufacture, Fourth Military Medical University, 145th West Changle Road, Xi’an, 710032 Shaanxi People’s Republic of China; 2 Shaanxi Key Laboratory of Free Radical Biology and Medicine, The Ministry of Education Key Laboratory of Hazard Assessment and Control in Special Operational Environments, Fourth Military Medical University, Xi’an, People’s Republic of China; 3 Department of Hepatobiliary Surgery, Xijing Hospital, Fourth Military Medical University, Xi’an, Shaanxi People’s Republic of China; 4 Department of Cell Biology, National Translational Science Center for Molecular Medicine, Fourth Military Medical University, Xi’an, Shaanxi People’s Republic of China

**Keywords:** Melatonin, Cellular senescence, Cell aging, Autophagy, Cell expansion, Translational medicine

## Abstract

**Background:**

Stem cells that have undergone long-term ex vivo expansion are most likely functionally compromised (namely cellular senescence) in terms of their stem cell properties and therapeutic potential. Due to its ability to attenuate cellular senescence, melatonin (MLT) has been proposed as an adjuvant in long-term cell expansion protocols, but the mechanism underlying MLT-induced cell rejuvenation remains largely unknown.

**Methods:**

Human periodontal ligament stem cells (PDLSCs) were isolated and cultured ex vivo for up to 15 passages, and cells from passages 2, 7, and 15 (P2, P7, and P15) were used to investigate cellular senescence and autophagy change in response to long-term expansion and indeed the following MLT treatment. Next, we examined whether MLT could induce cell rejuvenation by restoring the autophagic processes of damaged cells and explored the underlying signaling pathways. In this context, cellular senescence was indicated by senescence-associated β-galactosidase (SA-β-gal) activity and by the expression of senescence-related proteins, including p53, p21, p16, and γ-H2AX. In parallel, cell autophagic processes were evaluated by examining autophagic vesicles (by transmission electronic microscopy), autophagic flux (by assessing mRFP-GFP-LC3-transfected cells), and autophagy-associated proteins (by Western blot assay of Atg7, Beclin-1, LC3-II, and p62).

**Results:**

We found that long-term in vitro passaging led to cell senescence along with impaired autophagy. As expected, MLT supplementation not only restored cells to a younger state but also restored autophagy in senescent cells. Additionally, we demonstrated that autophagy inhibitors could block MLT-induced cell rejuvenation. When the underlying signaling pathways involved were investigated, we found that the MLT receptor (MT) mediated MLT-related autophagy restoration by regulating the PI3K/AKT/mTOR signaling pathway.

**Conclusions:**

The present study suggests that MLT may attenuate long-term expansion-caused cellular senescence by restoring autophagy, most likely via the PI3K/AKT/mTOR signaling pathway in an MT-dependent manner. This is the first report identifying the involvement of MT-dependent PI3K/AKT/mTOR signaling in MLT-induced autophagy alteration, indicating a potential of autophagy-restoring agents such as MLT to be used in the development of optimized clinical-scale cell production protocols.

**Supplementary Information:**

The online version contains supplementary material available at 10.1186/s13287-021-02322-9.

## Background

Postnatal mesenchymal stromal cells (MSCs) can be isolated from many adult human tissues, including but not limited to the bone marrow, adipose tissue, skin, articular cartilage, brain, and multiple dental tissues, such as the periodontium. Although MSCs have been demonstrated to be powerful therapeutics for various injuries and disorders in preclinical settings, one major impediment in many if not all current scalable protocols is that cellular senescence occurs during long-term culture and hence compromises the subsequent therapeutic effects of MSCs in the clinical setting [1–4]. In fact, how to expand quantity- and quality-assured cellular materials to be used in the clinical setting remains to be explored [5–7]. Successful cellular therapy requires a sufficient quantity of robust cells, while the production of cellular materials of interest at a clinical scale necessitates prolonged in vitro cell expansion. Therefore, it is essential to enhance our knowledge about the culture-caused cell aging process and indeed to identify new agents that can attenuate cellular senescence during long-term cell expansion.

Senescence-associated β-galactosidase (SA-β-gal) expression, reactive oxygen species (ROS) accumulation, DNA damage, mitochondrial dysfunction [8], metabolic alterations [9], epigenetic modifications [10], and/or telomerase/chromosome anomalies can be identified in aging cells; hence, all are indicative of senescence [11]. More importantly, accumulating evidence indicates that perturbed autophagy is more frequently found in senescent stem cells than in other cell types, suggesting a new target to attenuate cell aging [12, 13]. In fact, autophagy has long been believed to be an evolutionarily conserved catabolic process that plays a cytoprotective role in maintaining cellular homeostasis. In this context, recent investigations have demonstrated that autophagy downregulation could cause the accumulation of dysfunctional macromolecules and organelles as well as imbalanced proteostasis and amino acid pools, leading to impaired metabolic functions. Interestingly, the promotion of autophagy could attenuate senescence-related dysfunction. Similar to stem cells extracted from physiologically aged animal models, knockout of certain autophagy-related proteins in stem cells results in a decreased cell regeneration capacity [12, 14]. Therefore, it is reasonable to presume that autophagy-restoring agents should be able to be used in long-term and clinical-scale cell expansion protocols to maintain a younger state in cells.

Melatonin (MLT), *N*-acetyl-5-methoxytryptamine, is an endogenous hormone that was first recognized as an important modulator of circadian rhythms, regulating various physiological bioactivities [15–17]. Due to its remarkable antioxidative, antiapoptotic, immune-regulatory, and multilineage differentiation functions [18–21], MLT has been used in many biological fields. Although the underlying mechanism remains unknown, in vitro use of MLT demonstrated to prevent senescence of MSCs [22, 23], and its exogenous administration has been proven to be effective in prolonging longevity in several model organisms [24–26]. These findings indicate a close connection between MLT and cellular senescence. Although some methods, such as the treatment of stem cells with growth factors, the cultivation of cells in biomaterials mimicking in vivo cell environment and gene modification, alone or in combination, have been applied to try to retard cell senescence and maintain stemness in many cell production protocols, these methods cannot be widely used due to their high cost, technique complexity, and/or risks. Compared to expensive growth factors, complex biomaterials or intricate gene modification strategies, commercially manufactured MLT is a natural agent that has been safely used in preclinical and clinical settings for research and therapeutic purposes for decades. In this regard, MLT should be superior to other adjuvants in terms of safety, cost-effectiveness, and efficacy for large-scale cellular materials undergoing long-term expansion, although the comprehensive mechanisms are still under investigation. In regard to the therapeutic function of MLT, in vivo MLT application restored autophagic flux to impede cognitive decline and cardiac anomalies in Alzheimer’s disease (AD) animal models [27, 28]. More recently, MLT was demonstrated to downregulate PI3K/AKT in stem cells through the MLT receptor (MT) [29]. Given that the PI3K/AKT/mTOR pathway is a main signaling pathway of autophagy regulation [30, 31], we hypothesize that the mechanism underlying MLT-induced senescence attenuation is attributed to its regulation of cell autophagy and that MT/PI3K/AKT/mTOR signaling is likely to be an important pathway involved in this regulatory process.

To test our hypothesis, we isolated human periodontal ligament (PDL) stem cells (PDLSCs) using established protocols in present study and cells cultured ex vivo for up to 15 passages. Cells from passages 2, 7, and 15 (P2, P7, and P15) were used to assess cellular senescence and changes in autophagy levels caused by long-term expansion. Next, the cellular responses of P2, P7, and P15 cells to MLT treatment were evaluated to test the biological function of MLT in maintaining cell stemness and indeed its ability to restore autophagy. Finally, we screened and verified the potential signaling pathways involved in MLT-induced cell rejuvenation.

## Methods

### In vitro expansion of PDLSCs

Human PDLSCs were isolated from extracted third molars or orthodontic teeth from 6 donors (2 males, 4 females, aged 26.5 ± 6.3) admitted to the Department of Oral and Maxillofacial Surgery, School of Stomatology, Fourth Military Medical University. All donors signed the informed consent form in advance. Extracted teeth were examined in advance to exclude periodontitis and were immediately transferred into a centrifuge tube containing complete culture medium (α-minimum essential medium (α-MEM; Invitrogen, Carlsbad, USA) containing 100 U/mL penicillin (Invitrogen), 100 μg/mL streptomycin (Invitrogen), and 10% fetal bovine serum (FBS; Sijiqing, Hangzhou, China)) and taken to the laboratory. After repeatedly rinsing with sterile phosphate-buffered saline (PBS; Corning, USA), PDL tissue was gently scraped from the middle of the root surface. The scraped tissue was collected and digested in 3 mg/mL type I collagenase (Sigma-Aldrich, St. Louis, USA) at 37 °C for 1 h. After digestion, tissues were seeded in 6-well plates (Thermo Scientific, Carlsbad, USA) with complete culture medium. The medium was changed every 2 days until cells migrated from the tissue. We recognized these primary cells as P0 cells. When the P0 cells reached 80% confluence, they were digested and seeded into a 75 cm^2^ flask (Thermo Scientific). The cells cultured in the flask were designated as P1 cells. Then, the cells were cultured and passaged whenever they reached 80% confluence until P15. To eliminate the individual differences in cells from different donors, P2, P7, and P15 cells from the same donor were labeled and cryopreserved in liquid nitrogen using CELLSAVING (New Cell & Molecular Biotech, Suzhou, China) and thawed at the same time for the identification of MSC properties and further investigations.

### Characterization of PDLSCs in various passages (P2, P7, and P15)

#### Flow cytometry analysis

P2, P7, and P15 cells were digested, collected, and washed twice with PBS. Then, the cells were transferred into sterile Eppendorf tubes (Eppendorf, Hamburg, Germany) with at least 1 × 10^5^ cells/tube. The cells were incubated at 4 °C in the dark for 1 h with monoclonal antibodies against human CD90, CD105, CD146, CD34, CD45, or CD31 (all from eBioscience, San Diego, CA, USA) at a 1:1000 dilution, and cells incubated with PBS were used as the negative control. The immunophenotypes of the cells were assessed with a Beckman Coulter Epics XL cytometer (Beckman Coulter, Fullerton, CA, USA).

#### Colony formation assay

P2, P7, and P15 cells were seeded into 100-mm culture dishes (Invitrogen) at a density of 1 × 10^3^ cells/well. The medium (complete α-MEM) was refreshed every 2 days for 10 days. The cells were then rinsed twice, fixed with 4% paraformaldehyde (Servicebio, Wuhan, China) for 20 min, and stained with 0.1% toluidine blue (Sigma-Aldrich) for 15 min. The stained cells were rinsed to wash away extra-dye and then were observed under a stereomicroscope (Olympus Optical, Tokyo, Japan). Cell aggregates containing more than 50 cells were recognized as colonies.

#### Cell Counting Kit-8 (CCK-8) assay

P2, P7, and P15 cells were seeded into 96-well plates (Invitrogen) at a density of 1 × 10^3^ cells/well. The medium was refreshed every 2 days for 8 days. During the 8-day culture, 200 μL medium with 20 μL CCK-8 reagent (Dojindo Corporation, Tokyo, Japan) was added to each well at the same time every day, and the plate was incubated at 37 °C for 1 h; then, the absorbance at 450 nm was detected with a microplate reader (TECAN, Männedorf, Switzerland) to measure the proliferation ability of cells.

#### Cell differentiation assay

P2, P7, and P15 cells were seeded into 6-well dishes at a density of 5 × 10^5^ cells/well. After the cells reached 80% confluence, the medium was changed to osteo-inductive medium, adipo-inductive medium, or chondrogenic inductive medium (all from Cyagen, Guangzhou, China) to ascertain their alkaline phosphatase (ALP) activity and multilineage differentiation potential; the medium was refreshed every 2 days. Based on the methods reported previously and/or according to the manufacturer’s instructions, ALP staining (ALP staining kit, Beyotime Institute of Biotechnology, Nantong, China) and Alizarin red staining (kit from Sigma-Aldrich) were performed following osteo-induction for 7 and 21 days. In parallel, Oil red O staining (Cyagen) and Alcian blue staining (Cyagen) were performed following adipo-induction or chondrogenic induction for 21 days or 28 days. To quantitatively analyze the mineralized nodules after Alizarin red staining, the mineralized nodules were dissolved with 6% cetyl-pyridine for 15 min. Then, the absorbance at 570 nm was assessed with a microplate reader (TECAN). ALP quantitative analysis was performed using an alkaline phosphatase assay kit (Nanjing Jiancheng Bioengineering Institute, Nanjing China) according to the manufacturer’s instructions.

### Cellular senescence and autophagy levels of P2, P7, and P15 cells

Cryopreserved P2, P7, and P15 cells from the same donor were thawed at the same time and seeded for investigations. P2 cells were used as the control group, while P7 and P15 cells were used as senescent groups. To detect cell senescence features, SA-β-gal activity (see Section “SA-β-gal activity analysis”) and the expression of senescence-associated proteins p16, p21, p53, and γ-H2AX (see Section “Western blot analysis”) were evaluated. To investigate the autophagy level in P2, P7, and P15 cells, we first verified the existence of autophagic vesicles by transmission electron microscopy (see Section “Transmission electron microscopy”). Then, the autophagic flux in P2, P7, and P15 cells was detected. To determine changes in autophagic flux occurring before or after autolysosome formation, 1 μM bafilomycin A1 (Baf; MedChemExpress), an inhibitor of autophagosome-lysosome fusion, was added for 4 h before the tests. After mRFP-GFP-LC3 adenovirus transfection, P2, P7, and P15 cells were observed under a confocal microscope (see Section “Cell transfection”). Another marker of autophagic flux, LC3-II, was evaluated by flow cytometry analysis (see Section “Indirect immunofluorescent labeling and flow cytometry analysis”). The alteration of autophagy levels in P2, P7, and P15 cells was further confirmed by detecting the expression of autophagy-related proteins Atg7, p62, and Beclin-1 (see Section “Western blot analysis”).

### MLT concentration selection

To select a suitable concentration for cell treatment, 0, 10 nM, 100 nM, 1 μM, or 10 μM MLT (Sigma-Aldrich) was added to complete culture medium and incubated with P15 cells for 24 h. Cell viability (see Section “CCK-8 assay”) and the expression of cellular senescence-associated genes and autophagy-associated proteins (see Sections “qRT-PCR analysis” and “Western blot analysis”) in response to MLT treatment at various concentrations. An MLT concentration that enhanced cell viability, attenuated cellular senescence, and activated autophagy was selected for cell treatment in other investigations.

### Effects of MLT treatment on senescent cells

Based on the selected MLT concentration, we investigated the cell response (rejuvenation) and autophagy level of P7 and P15 cells following MLT treatment; P2 cells were used as the control. To investigate the changes in cell proliferation in response to MLT treatment, MLT was added to complete α-MEM from day 1 to day 8 to perform the CCK-8 assay (see Section “CCK-8 assay”). To evaluate the pro-osteogenesis effect of MLT, MLT was added during osteo-induction from day 1 to day 21 at a concentration of 1 μM, as selected based on dose-response assays. The osteogenic differentiation potential of P15 cells with or without MLT treatment was evaluated by Alizarin red staining (see Section “Cell differentiation assay”) and ALP staining (see Section “ALP staining”), and osteogenesis-related protein expression (RUNX2 and OCN) was determined by Western blot analysis (see Section “Western blot analysis”). Cell senescence and autophagy were assessed after MLT treatment for 24 h. The alteration of cell senescence in response to MLT treatment was evaluated by SA-β-gal activity (see Section “SA-β-gal activity analysis”) and the expression of senescence-associated proteins p16, p21, p53, and γ-H2AX (see Section “Western blot analysis”). The changes in autophagy were assessed in terms of autophagic flux and the expression of autophagy-related proteins. 1 μM Baf was added to determine whether MLT altered autophagy by promoting the initiation of autophagic flux or by inhibiting the degradation of autophagosomes. Fluorescently labeled autophagic vesicles were observed in mRFP-GFP-LC3 adenovirus-transfected cells under a confocal microscope (see Section “Cell transfection”), and the amount of LC3-II was detected by flow cytometry analysis (see Section “Indirect immunofluorescent labeling and flow cytometry analysis”) to assess the autophagic flux after MLT treatment. The expression of the autophagy-related proteins Atg7, p62, and Beclin-1 was evaluated by Western blotting (see Section “Western blot analysis”).

### Effects of autophagy inhibition on MLT-mediated cellular rejuvenation

To determine the role of autophagy in MLT-mediated rejuvenation, 5 mM 3-methyladenine (3-MA, MedChemExpress, New Jersey, USA) was used as an autophagy inhibitor to counteract MLT-mediated autophagy induction. P15 cells were used as study subjects and divided into four groups. P15 cells incubated in complete α-MEM without any treatment were used as the control (named the CON group). P15 cells incubated with MLT (named the MLT group) and 3-MA (named the 3-MA group) separately or together (named the MLT + 3-MA group) for 24 h were used for the assessment of cell senescence and autophagy.

### Role of MT in MLT-mediated cellular rejuvenation and autophagy induction

To determine whether MT was involved in the MLT-mediated effect, the expression of MT in PDLSCs was investigated by Western blotting (see Section “Western blot analysis”). Then, 1 μM luzindole (LUZ; MedChemExpress), a nonselective antagonist of MLT receptors, was added to inhibit the interaction between MLT and MT. P15 cells were used as study subjects and incubated in complete α-MEM with MLT and LUZ alone or together for 24 h to assess the alterations in cell autophagy and senescence when MT was inhibited. P15 cells incubated in complete α-MEM without any treatment were used as the control group.

### Role of PI3K/AKT/mTOR signaling in MLT-mediated cellular rejuvenation and autophagy induction

To investigate the possible pathway involved in MT-dependent autophagy, LUZ was added to assess the total protein expression of PI3K, AKT, and mTOR and the expression of p-PI3K, p-AKT, and p-mTOR by Western blotting (see Section “Western blot analysis”) after the interaction between MLT and MT was inhibited. Then, the involvement of the PI3K/AKT/mTOR signaling pathway was further studied by treating the cells with SC79 (PI3K/AKT activator) and MHY1485 (mTOR activator) (both purchased in MedChemExpress) in complete α-MEM for 24 h. After the given treatment, the alterations in cell senescence and autophagy were assessed. Each reagent was first dissolved in DMSO (Sigma-Aldrich) to form a storage solution at a concentration of 10 mM and then diluted to the final applied concentration in basal medium before use.

#### SA-β-gal activity analysis

To detect cellular senescence, SA-β-gal activity was evaluated. Cells were seeded into 6-well dishes at a density of 1 × 10^5^ cells/well. After the cells reached 50% confluence, SA-β-gal activity was detected using a senescence β-galactosidase staining kit (Cell Signaling Technology (CST), MA, USA) according to the manufacturer’s instructions. SA-β-gal-positive cells were dyed blue, observed, and calculated based on three randomly selected bright fields. The percentage of SA-β-gal-positive cells among all cells was analyzed.

#### Transmission electron microscopy

To detect autophagic vesicles, cells were fixed using 3% (w/v) glutaraldehyde for at least 48 h at 4 °C and postfixed with osmium tetroxide. The samples were dehydrated in a graded series of alcohol concentrations, embedded in epoxy resin, and then sectioned. The autophagic vesicles in cells were observed by transmission electron microscopy (Hitachi, Tokyo, Japan).

#### Cell transfection

To visualize autolysosomes/autophagosomes under a laser confocal microscope, PDLSCs were transfected with adenovirus mRFP-GFP-LC3 (HanBio Technology, Shanghai, China) prior to use. Briefly, the cells were seeded into confocal 24-well dishes (Xinyou Biotechnology, Hangzhou, China) at a density of 5 × 10^4^ cells/well. After the cells adhered and reached 50% confluence, they were transfected with mRFP-GFP-LC3 adenovirus according to the manufacturer’s instructions. The medium was renewed 2 h after transfection to remove extra adenovirus. Pharmaceutical treatments were conducted 12 h after transfection. After the respective treatments, the transfected cells were fixed with 4% paraformaldehyde (Servicebio) and observed under an Olympus FV1000 laser confocal microscope (Tokyo, Japan). Both red and yellow puncta were counted and analyzed in the merged image based on three randomly selected fields.

#### Indirect immunofluorescent labeling and flow cytometry analysis

The amount of LC3-II, which is recognized as a marker that is altered during autophagic flux, was evaluated by indirect immunofluorescent labeling for GFP-LC3-II and subsequent flow cytometry analysis. The cells were fixed and permeabilized with the Fixation/Permeabilization Solution Kit (BD Biosciences Pharmingen, San Diego, USA) for 20 min at 4 °C. After rinsing twice, the cells were incubated with an LC3B-specific antibody (rabbit polyclonal, ProteinTech, Illinois, USA, # 18725-1-AP) at a 1:200 dilution at 4 °C overnight. Then, the cells were rinsed three times to remove additional primary antibody before incubation at room temperature in the dark for 2 h with goat anti-rabbit DyLight 488 IgG (1:200, AntiProtech Inc., California, USA). The labeled cells were analyzed using a Beckman Coulter Epics XL cytometer (Beckman Coulter).

#### RNA extraction and quantitative real-time polymerase chain reaction (qRT-PCR)

Total RNA was extracted using the EZNA Total RNA Kit II (OMEGA Biotek, Inc., GA, USA) according to the manufacturer’s instructions. Extracted total RNA was reverse transcribed to cDNA using the PrimeScript RT Reagent Kit (TaKaRa, Shiga, Japan) as instructed. qRT-PCR analysis was performed using the SYBR Premix Ex Taq II Kit (TaKaRa), and the results were assessed with a CFX96 Real-time RT-PCR System (Bio-Rad, Hercules, CA, USA). β-Actin was used as the endogenous control to normalize the expression levels. The resulting amplification and melt curves were analyzed to ensure the identity of the specific PCR product. Threshold cycle values were used to calculate the fold change in the transcript levels by using the 2^−ΔΔCt^ method. The primers used for qRT-PCR were as follows: β-actin: sense 5′-TGGCACCCAGCACAATGAA-3′ and antisense 5′-CTAAGTCATAGTCCGCCTAGAAGCA-3′; p53: sense 5′-CAGCACATGACGGAGGTTGT-3′ and antisense 5′-TCATCCAAATACTCCACACGC-3′; p21: sense 5′-TGTCCGTCAGAACCCATGC-3′ and antisense 5′-AAAGTCGAAGTTCCATCGCTC-3′; and p16: sense 5′-GAAGAAAGAGGAGGGGCTG-3′ and antisense 5′-GCGCTACCTGATTCCAATTC-3′.

#### Western blot analysis

Prepared cells were lysed in RIPA buffer (Beyotime) supplemented with phosphatase inhibitors (Sigma-Aldrich). The protein concentrations were measured using a bicinchoninic acid (BCA) assay kit (Solarbio, Beijing, China). Protein samples were separated by sodium dodecyl sulfate-polyacrylamide gel electrophoresis (SDS-PAGE) (Beyotime). The concentration of the applied SDS-PAGE gel was selected according to the molecular weight of the proteins. That is, 15% gels were used to assess OCN, LC3, p16, γ-H2AX, and p21; 10% gels were used to assess p-PI3K, PI3K, p-AKT, AKT, Atg7, Beclin-1, p53, RUNX2, and GAPDH; and 6% gels were used to assess p-mTOR and mTOR. The proteins were transferred onto PVDF membranes (Millipore, Billerica, MA, USA). After blocking with 5% nonfat milk at room temperature for 1 h, the membranes were incubated with primary antibodies at 4 °C overnight and with the corresponding horseradish peroxidase (HRP)-conjugated secondary antibody (1:2000; goat anti-rabbit IgG, CST, #7074; goat anti-mouse IgG, CST, #7076) for 2 h at room temperature the next day. Finally, the blots were detected using enhanced chemiluminescence substrate (ECL kit, Millipore). The phosphorylated protein level was normalized to the corresponding total protein level. GAPDH was used as the housekeeping gene for internal normalization. ImageJ software was used to analyze protein bands. For Western blotting, we used primary antibodies against p16 (1:1000; ProteinTech, #10883-1-AP), p21 (1:1000; ProteinTech, #10355-1-AP), p53 (1:1000; Abcam, Cambridge, Britain, ab26), γ-H2AX (1:1000; Abcam, ab229914), LC3B (1:1000; ProteinTech, #14600-1-AP), p62 (1:1000; ProteinTech, #18420-1-AP), Atg7 (1:1000; ProteinTech, #10088-2-AP), Beclin-1 (1:1000; ProteinTech, #11306-1-AP), OCN (1:1000; Abcam, ab93876), RUNX2 (1:1000; CST, #12556), MTNR1A (1:1000; ProteinTech, #Ag24198) PI3K (1:1000; CST, #4257), p-PI3K (Tyr458) (1:1000; CST, #4228), AKT (1:1000; CST, #4691), p-AKT (Ser473) (1:1000; CST, #4060), mTOR (1:1000; CST, #2983), and p-mTOR (Ser2448) (1:1000; CST, #5536).

### Statistical analysis

The PDLSCs compared before and after long-term passaging were from identical donors. All assays were performed at least three times independently. GraphPad Prism 7 software was employed for statistical analysis. Differences between groups were assessed by one-way analysis of variance (one-way ANOVA) followed by Tukey’s multiple comparisons tests, Sidak’s multiple comparisons tests, or Dunnett’s multiple comparisons tests. The results are presented as the mean and standard deviation (mean ± SD). All experiments were repeated in PDLSCs from at least 3 different donors. Statistical significance was expressed as *p* < 0.05 (*), *p* < 0.01 (**), *p* < 0.001 (***), or *p* < 0.0001 (****).

## Results

### Long-term passaging causes cellular senescence

**Fig. 1:**
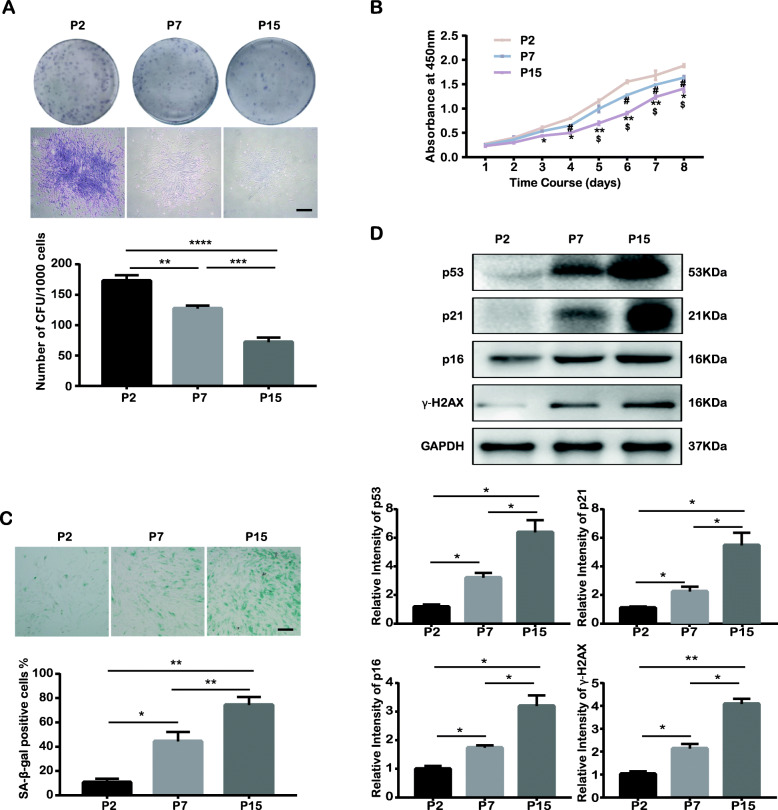
Long-term ex vivo culture causes senescence in human PDLSCs. Human PDLSCs were cultured and passaged in vitro; across the ex vivo expansion stage, passage 2 (P2), passage 7 (P7), and passage 15 (P15) cells were collected for subsequent studies. *p < 0.05, **p < 0.01, ***p < 0.001, and ****p < 0.0001 represent significant differences between the indicated columns. **a** The colony-forming ability of PDLSCs changed during long-term ex vivo expansion (top: general views of the cell colonies formed by P2, P7, and P15 cells and a representative single colony from the top panel (scale bar = 100 μm); bottom: quantitative analysis of cell colonies formed by P2, P7, and P15 cells). **b** Proliferative activity of P2, P7, and P15 cells in terms of CCK-8 assay (P2 vs. P15: *p < 0.05 and **p < 0.01; P2 vs. P7: ^#^*p* < 0.05; P7 vs. P15: ^$^*p* < 0.05). **c** SA-β-gal in P2, P7, and P15 cells (top: representative images of SA-β-gal expression in P2, P7, and P15 cells (immunocytochemical staining, positively stained cells were dyed in blue; scale bar = 100 μm); bottom: percentage of SA-β-gal-positive cells among all cells and quantitative analysis). **d** Cell senescence-related proteins in P2, P7, and P15 cells (top: protein levels of p53, p21, p16, and γ-H2AX as determined by Western blot assay; bottom: quantitative analysis of p53, p21, p16, and γ-H2AX expression in P2, P7, and P15 cells)

PDLSCs were successfully isolated from PDL tissues from 6 donors (2 males, 4 females, aged 26.5 ± 6.3) and were expanded in vitro for 15 passages according to the methods described in the corresponding section. Here, we chose P2 cells as the control group and P7 and P15 cells from the same donor as study subjects to investigate the alterations that occur alongside long-term ex vivo expansion. First, we detected the cell surface markers of MSCs by flow cytometry analysis. We found that all P2, P7, and P15 cells positively expressed MSC markers, including CD90, CD105, and CD146, while the endothelial cell marker CD31 and hematopoiesis-related markers CD34 and CD45 were not expressed (Fig. S1a). Then, the differentiation potential of P2, P7, and P15 cells towards cartilage, adipose, and osseous tissue was investigated by Alcian blue, Oil red O, Alizarin red, and ALP staining assays following the given protocols. The stained acidic polysaccharides, lipid droplets, and mineralized nodes suggested that P2, P7, and P15 cells all had multilineage differentiation potential for processes including chondrogenesis, adipogenesis, and osteogenesis (Fig. S1b–d and f). However, quantitative analysis of Alizarin red staining and ALP activity suggested that the osteogenesis potential of P15 cells was significantly decreased compared to that of P2 cells (Fig. S1e and g). Furthermore, as indicated by the colony formation assay and CCK-8 assay, the cells from all passages were proven to possess the properties of colony formation and proliferation. However, the proliferation rate significantly declined with long-term passaging (Fig. 1a, b). Accordingly, SA-β-gal activity, which reflects the extent of cellular senescence [32], was markedly higher in P15 cells (Fig. 1c). To further verify cellular senescence after in vitro expansion, the expression of several senescence-associated proteins, including p16, p21, p53, and γ-H2AX, was investigated. p16, p21, and p53 are recognized as cell cycle regulatory proteins, and their increased expression often indicates cell cycle dysregulation. γ-H2AX, which forms discrete nuclear foci when phosphorylated, sensitively indicates DNA double-strand break damage. Western blotting indicated that the expression of these senescence-related proteins was increased after long-term passaging (Fig. 1d). In summary, senescence-associated features, including proliferation inhibition, decreased osteogenesis potential, increased SA-β-gal activity, cell cycle arrest, and DNA damage, were observed as the passage number increased (from P2 to P7 to P15, with the most prominent effects in P15 cells), suggesting that long-term passaging causes cellular senescence.

### Cell autophagy is impaired after long-term passaging

**Fig. 2:**
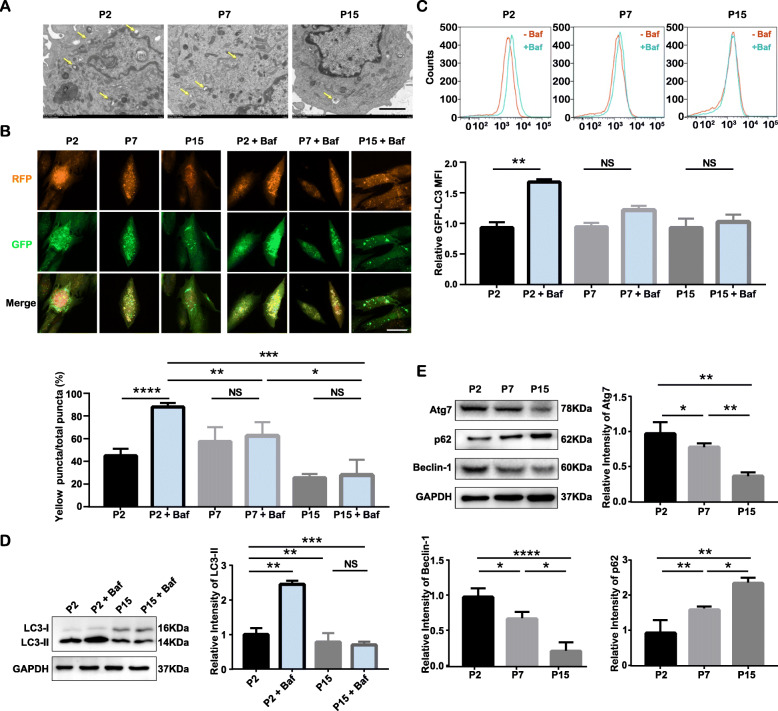
Long-term ex vivo culture causes defective autophagy in human PDLSCs**.** *p < 0.05, **p < 0.01, ***p < 0.001, and ****p < 0.0001 represent significant differences between the indicated columns, while NS represents no significant difference. **a** Electron microscopy images of P2, P7, and P15 cells. Arrowheads indicate autophagic vesicles. Scale bar = 2 μm. **b** Presence of autophagosomes (yellow) and their maturation into autolysosomes (red) in adenovirus mRFP-GFP-LC3-transfected cells (P2, P7, P15) with or without Baf treatment (top: representative confocal images, scale bar = 50 μm, ×200 magnification; bottom: quantitative analysis of the percentage of yellow puncta out of the total puncta based on confocal examination). **c** GFP–LC3 fluorescence levels in P2, P7, and P15 cells before and after Baf treatment (top: representative flow cytometry graphs; bottom: quantitative analysis of the MFI of GFP–LC3). **d** LC3-II levels in P2 and P15 cells before and after Baf treatment (left: protein levels of LC3-II as determined by Western blot assay; right: quantitative analysis of LC3-II expression in cells). **e** Expression of cell autophagy-related proteins in P2, P7, and P15 cells as determined by Western blot assay; quantitative analysis of Atg7, p62, and Beclin-1 expression

The alterations in autophagy levels after long-term passaging were evaluated in terms of the observation of autophagic vesicle formation, autophagic flux, and autophagy-related protein expression. We first observed autophagic vesicles in P2, P7, and P15 cells by transmission electron microscopy. According to the observations, double-layered autophagic vesicles existed in all P2 and P7 cells, but were scarcely observed in P15 cells (Fig. 2a). The observed double-layered autophagic vesicles could be autophagosomes or autolysosomes, but it was hard to distinguish the type merely via the observation by transmission electron microscopy. To distinguish autophagosomes from autolysosomes and to detect the proceeding autophagic flux in P2, P7, and P15 cells, the mRFP-GFP-LC3 adenovirus reporter was transfected into P2, P7, and P15 cells. Autophagy is a consecutive process that includes autophagosome formation, maturation, fusion with lysosomes to form autolysosomes, and degradation. The process from autophagosome formation to degradation is termed autophagic flux. Generally, LC3-II is currently the most widely used marker for autophagosome and autophagic flux detection [33]; LC3-II is located on both the outer and inner membranes of autophagosomes, and its expression level decreases as autophagosomes are fused with and subsequently degraded by lysosomes. In mRFP-GFP-LC3 adenovirus-transfected cells, both RFP and GFP were expressed along with LC3. When autophagosomes did not fuse with lysosomes, they displayed yellow puncta formed by the overlay of red and green puncta. When autophagosomes fused with lysosomes to form autolysosomes, the inner compartment, which has a low pH (approximately 4 ~ 5) quenches the fluorescence of GFP, and thus autolysosomes can be visualized as red puncta. After long-term passaging, the number of red puncta (autolysosomes) prominently declined in P15 cells (Fig. 2b). To further verify whether the decline in autolysosomes in senescent cells was caused by inhibited formation or increased degradation, Baf, an inhibitor of lysosome formation, was added 4 h before the tests. After Baf supplementation, P2 cells were saturated with yellow puncta (non-fused autophagosomes) because of the failure of autolysosome formation; however, the alteration of the accumulation of yellow puncta was attenuated in P7 and P15 cells, suggesting that the decreased volume of autophagic vesicles in cells after long-term expansion saturation did not result from increased degradation but from blocked autophagic flux (Fig. 2b).

We also used flow cytometry analysis and Western blotting to assess the amount of LC3-II to verify the blockage of autophagic flux after long-term expansion. There were no significant differences in the amount of LC3-II before and after Baf treatment in P7 and P15 cells, while LC3-II significantly accumulated in P2 cells after Baf treatment (Fig. 2c, d). Since the amount of LC3-II could reflect the number of autophagosomes, these results indicate the inhibition of autophagosome formation in the cells after long-term expansion. As indicated by Western blotting, the expression of autophagy-related proteins, including Atg7 and Beclin-1, decreased after long-term expansion, while the expression of the autophagy substrate indicator p62 increased with PDLSC senescence (Fig. 2e), altogether indicating the impaired autophagy in senescent cells caused by long-term expansion.

### MLT ameliorates PDLSC senescence and restores autophagy

To investigate the effect of MLT on senescent PDLSCs, MLT was added to the culture medium. First, the optimal MLT concentration was selected according to dose that yielded the most viable PDLSCs. P15 cells were treated with 0, 10 nM, 100 nM, 1 μM, or 10 μM MLT for 24 h. According to the CCK-8 assay, cells treated with 1 μM MLT showed the highest viability (Fig. S2a). Consistently, as indicated by Western blotting, the expression of autophagy-related proteins, including Atg7, Beclin-1, and LC3-II, was significantly higher, while that of p62 was lower in cells treated with 1 μM MLT than in those treated with other doses of MLT; these features collectively suggest a higher autophagy level in the 1 μM MLT-treated group (Fig. S2b and c). The expression of senescence-related proteins was evaluated by qRT-PCR. The expression of p16, p21, and γ-H2AX was lower in the 1 μM MLT group, while the expression of p53 showed no significant difference in the 100 nM and 1 μM MLT groups (Fig. S2d). To achieve optimal cell viability, better autophagy restoration and rejuvenation effects, we chose 1 μM MLT for further studies.

**Fig. 3:**
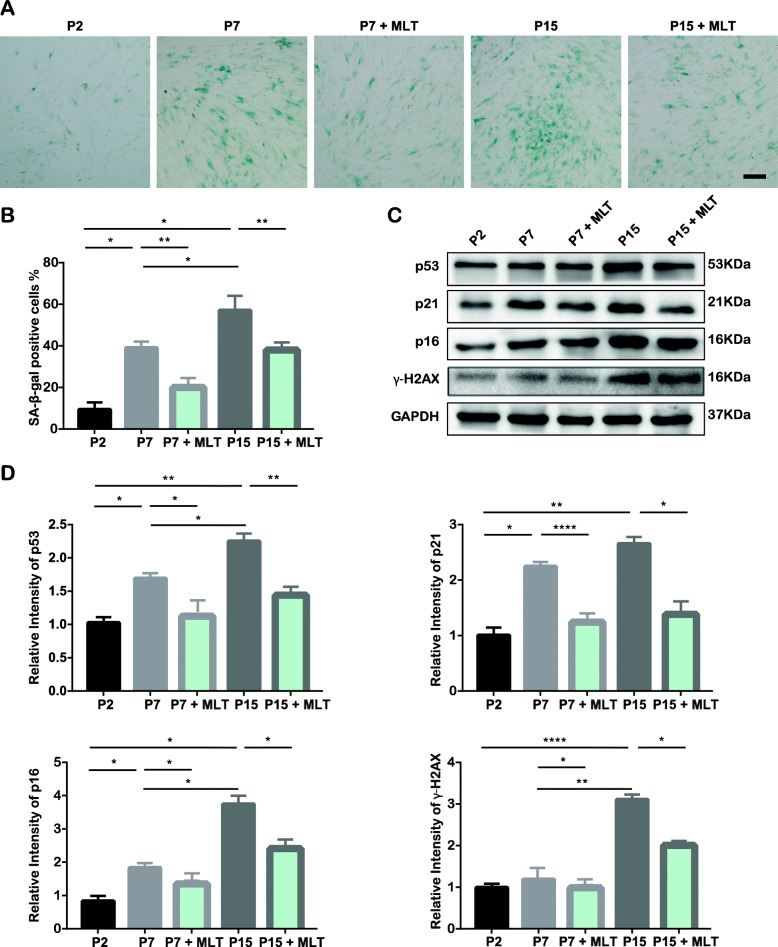
MLT rescues cells from long-term ex vivo passaging-induced senescence. (MLT-treated P2, P7, and P15 cells were termed P2 + MLT, P7 + MLT, and P15 + MLT, respectively). *p < 0.05, **p < 0.01, and ****p < 0.0001 represent significant differences between the indicated columns. **a** Representative images of SA-β-gal expression in P2, P7, and P15 cells (immunocytochemical staining, positively stained cells were dyed in blue; scale bar = 100 μm). **b** Percentage of SA-β-gal-positive cells among all cells and quantitative analysis. **c** Cell senescence-related protein expression in P2, P7, and P15 cells as determined by Western blot assay. **d** Quantitative analysis of p53, p21, p16, and γ-H2AX expression in P2, P7, and P15 cells

After treatment with MLT, SA-β-gal staining was employed to investigate the effect of MLT on cellular senescence. The proportion of SA-β-gal-positive cells was increased in P7 and P15 cells compared to P2 cells, but this effect was drastically reduced after MLT treatment (Fig. 3a, b). Consistent with the SA-β-gal staining results, the Western blotting results indicated that the increase in the expression of p16, p21, and p53 in P7 and P15 cells declined after MLT treatment (Fig. 3c, d). According to the CCK-8 assay, MLT could also promote the proliferation of P15 cells (Fig. S3a). For the alteration in osteogenic potential, MLT was added to the osteo-inductive microenvironment from day 1 to day 21. P2 cells undergoing osteogenic induction were used as the control group. Alizarin red and ALP staining assays indicated that MLT treatment promoted osteogenic differentiation in P15 cells (Fig. S3b–e). This result was further supported by the increased expression of osteoblast differentiation-related transcription factors RUNX2 and OCN, as indicated by Western blotting (Fig. S3f-h). These results collectively suggest the rejuvenation effect of MLT in senescent PDLSCs.

**Fig. 4:**
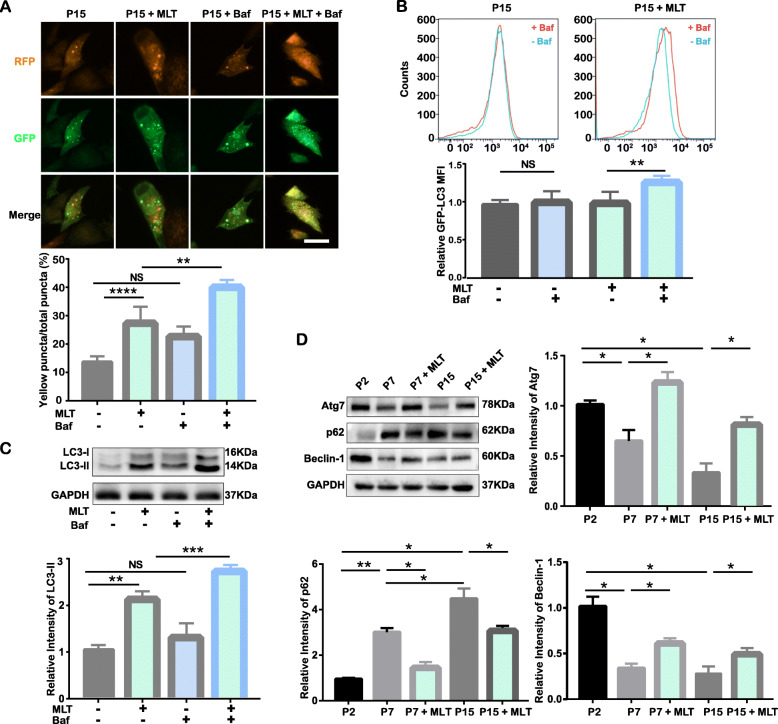
MLT restores autophagy in cells undergoing long-term ex vivo passaging. (MLT-treated P2, P7, and P15 cells were termed P2 + MLT, P7 + MLT, and P15 + MLT, respectively)**.** *p < 0.05, **p < 0.01, ***p < 0.001, and ****p < 0.0001 represent significant differences between the indicated columns, while NS represents no significant difference. **a** Presence of autophagosomes (yellow) and their matured form, autolysosomes (red), in mRFP-GFP-LC3 adenovirus-transfected cells with or without MLT treatment (top: representative confocal images, scale bar = 50 μm, ×200 magnification; bottom: quantitative analysis of the percentage of yellow puncta out of the total puncta based on confocal examination). **b** GFP-LC3 fluorescence levels in P15 and MLT-treated P15 cells before and after Baf treatment (top: representative flow cytometry graphs; bottom: quantitative analysis of the MFI of GFP–LC3). **c** LC3-II levels in P2 and P15 cells before and after Baf treatment (top: protein levels of LC3-II as determined by Western blot assay; bottom: quantitative analysis of cell LC3-II expression). **d** Expression of cell autophagy-related proteins in P2, P7, MLT-treated P7, P15, and MLT-treated P15 cells as determined by Western blot assay and quantitative analysis of Atg7, p62, and Beclin-1 expression

To detect the alteration in autophagic flux after MLT treatment, mRFP-GFP-LC3 adenovirus was transfected into P15 cells 24 h before MLT treatment. MLT was supplemented with culture medium for 24 h, and Baf was added 4 h before the tests. As shown in the merged image, the number of both red puncta and total puncta was elevated after MLT treatment, while Baf treatment further enhanced autophagosome accumulation (indicated by yellow puncta) in P15 cells treated with MLT (Fig. 4a). These observations suggest that MLT promotes the accumulation of both autophagosomes and autolysosomes, inferring propelled autophagic flux, in long-term expansion-induced senescent cells. As evidenced by Western blotting and flow cytometry, MLT increased the expression of LC3-II in P15 cells, and this effect was even more pronounced when cells were incubated with Baf, suggesting that the impairment of autophagic flux in P15 cells and could be attenuated after MLT treatment (Fig. 4b, c). Similarly, as verified by Western blotting, the decreases in the expression of Atg7 and Beclin-1 in P7 and P15 cells was significantly elevated after MLT treatment, while the accumulated p62 were significantly attenuated (Fig. 4d). These results collectively suggest that MLT treatment can restore autophagy in senescent cells.

In short, MLT treatment can restore autophagy and attenuate senescence in cells undergoing long-term expansion, but the relationship between the MLT-mediated rejuvenation effect and autophagy restoration needs to be further investigated.

### Inhibition of autophagy deteriorates cellular senescence

**Fig. 5:**
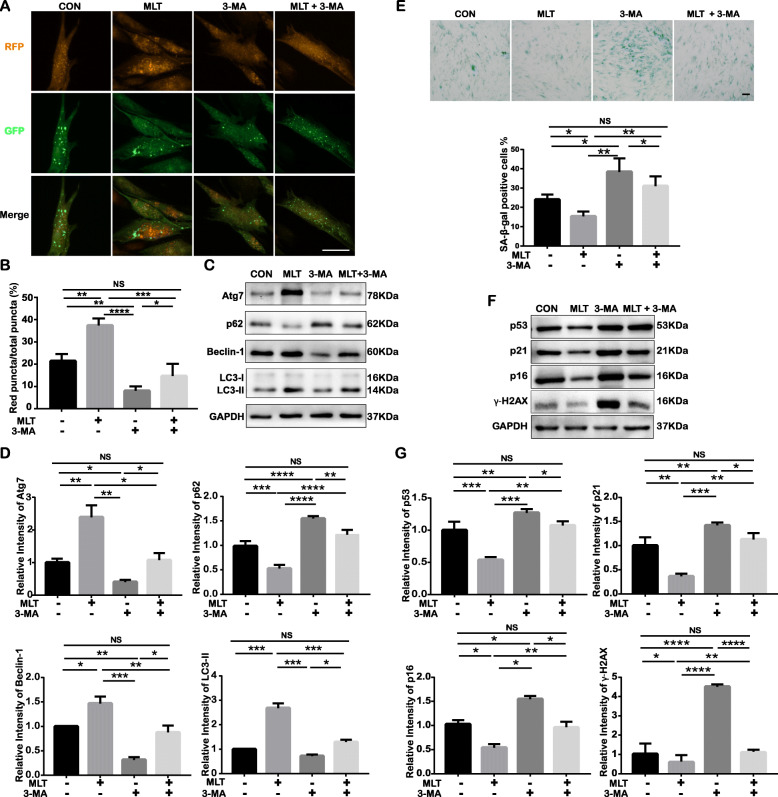
Downregulation of autophagy via an autophagy inhibitor deteriorated cell senescence. (CON: P15 cells without any treatment; MLT: MLT-treated P15 cells; 3-MA: 3-MA-treated P15 cells; MLT + 3-MA: MLT plus 3-MA–treated P15 cells**).** *p < 0.05, **p < 0.01, ***p < 0.001, and ****p < 0.0001 represent significant differences between the indicated columns, while NS represents no significant difference. **a** Representative confocal images of autophagosomes (yellow) and their matured form, autolysosomes (red), in mRFP-GFP-LC3 adenovirus-transfected P15 cells with or without MLT/3-MA treatment (scale bar = 50 μm, ×200 magnification). **b** Quantitative analysis of the percentage of red puncta out of the total puncta based on confocal examination. **c** Expression of cell autophagy-related proteins Atg7, p62, Beclin-1, and LC3-II in P15 cells with or without MLT/3-MA treatment as determined by Western blot assay. **d** Quantitative analysis of Atg7, p62, Beclin-1, and LC3-II expression in P15 cells with or without MLT/3-MA treatment. **e** SA-β-gal in P15 cells with or without MLT/3-MA treatment (top: representative images of SA-β-gal expression in terms of immunocytochemical staining; positively stained cells were dyed blue; scale bar = 100 μm; bottom: percentage of SA-β-gal-positive cells among all cells and quantitative analysis). **f** Cell senescence-related protein expression in P15 cells with or without MLT/3-MA treatment as determined by Western blot assay. **g** Quantitative analysis of p53, p21, p16, and γ-H2AX expression in P15 cells with or without MLT/3-MA treatment

To explore whether the rejuvenation effect mediated by MLT occurs through autophagy regulation, we cotreated P15 cells with MLT and the widely used autophagy inhibitor 3-MA to reverse the rescued autophagy. After treatment with MLT and 3-MA separately or simultaneously for 24 h, autophagy in P15 cells was evaluated in terms of the number of autophagic vesicles and the expression of autophagy-related proteins. As verified by mRFP-GFP-LC3 adenovirus transfection, the decreased number of red puncta (autolysosomes) and yellow puncta (non-fused autophagosomes) after 3-MA treatment suggested that 3-MA could further suppress autophagy in senescent cells and prevent MLT-induced autophagy (Fig. 5a, b). Similarly, Western blot analysis showed that the decreases in the expression of autophagy-related proteins, including Atg7, Beclin-1, and LC3-II, and the accumulation of autophagy substrate p62 in the 3-MA treatment group compared to the control group, suggesting that 3-MA could potently inhibit cell autophagy; the comparison between the MLT treatment group and the MLT and 3-MA cotreatment group suggested that 3-MA treatment also abrogated the restoration of autophagy mediated by MLT (Fig. 5c, d). These results collectively indicate that 3-MA could successfully impede MLT-induced autophagy restoration.

With the inhibition of autophagy caused by 3-MA, the senescence-associated changes were more pronounced compared to the control group. Even in the presence of MLT, 3-MA treatment increased the number of SA-β-gal-positive cells (Fig. 5e). Accordingly, the expression of the senescence-related proteins p16, p21, p53, and γ-H2AX further increased with 3-MA treatment (Fig. 5f, g), indicating increased cell cycle arrest and DNA damage along with impaired autophagy. The MLT-mediated rejuvenation effect was also suppressed by 3-MA treatment. Taken together, these data indicate that the loss of autophagy caused by 3-MA further exacerbates senescence-associated features and inhibits MLT-induced amelioration of senescence, suggesting that the MLT-mediated rejuvenation effect is associated with the restoration of autophagy.

### MT is involved in MLT-mediated autophagy regulation

**Fig. 6:**
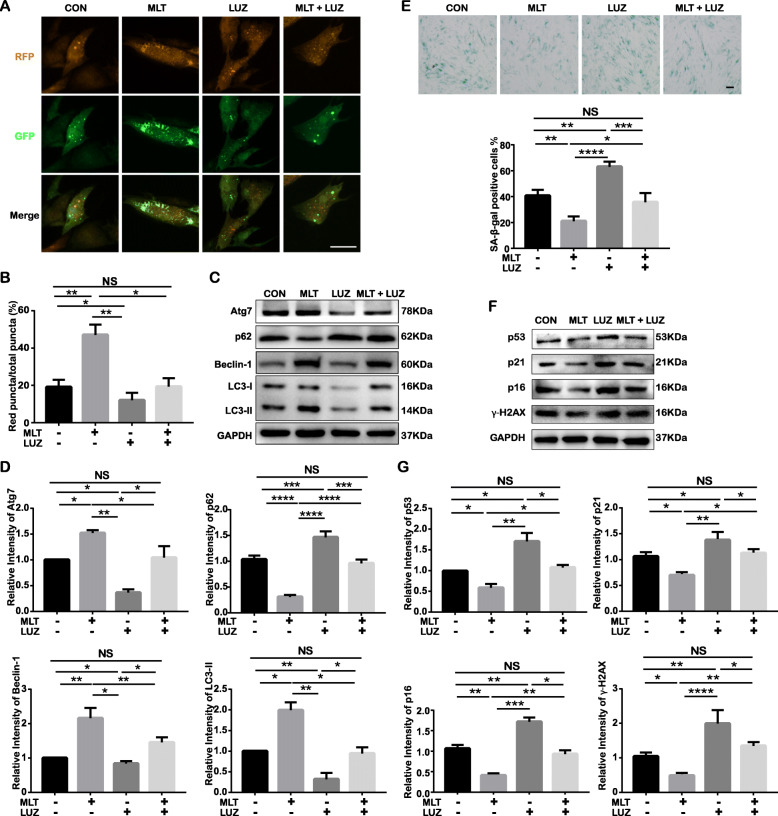
MT is involved in MLT-mediated autophagy regulation in cells undergoing long-term ex vivo passaging. (CON: P15 cells without any treatment; MLT: MLT-treated P15 cells; LUZ: LUZ-treated P15 cells; MLT + LUZ: MLT plus LUZ–treated P15 cells). *p < 0.05, **p < 0.01, ***p < 0.001, and ****p < 0.0001 represent significant differences between the indicated columns, while NS represents no significant difference. **a** Representative confocal images of autophagosomes (yellow) and their matured form, autolysosomes (red), in mRFP-GFP-LC3 adenovirus-transfected P15 cells with or without MLT/LUZ treatment (scale bar = 50 μm, ×200 magnification). **b** Quantitative analysis of the percentage of red puncta out of the total puncta based on confocal examination. **c** Cell autophagy-related proteins Atg7, p62, Beclin-1, and LC3-II were analyzed by Western blot assay. **d** Quantitative analysis of Atg7, p62, Beclin-1, and LC3-II expression in P15 cells with or without MLT/LUZ treatment. **e** SA-β-gal in P15 cells with or without MLT/LUZ treatment (top: representative images showing SA-β-gal expression by immunocytochemical staining; positively stained cells were dyed blue, scale bar = 100 μm; bottom: percentage of SA-β-gal-positive cells among all cells and quantitative analysis). **f** Cell senescence-related protein expression in P15 cells with or without MLT/LUZ treatment as determined by Western blot assay. **g** Quantitative analysis of p53, p21, p16, and γ-H2AX expression in P15 cells with or without MLT/LUZ treatment

To determine whether MLT-mediated autophagy regulation occurs in a receptor-dependent pathway, we first detected the expression of MT in PDLSCs. Western blotting indicated that P2, P7, and P15 cells all positively expressed MT, but there was no significant difference between the expression levels in these cells (Fig. S4a and b). Next, the cells were treated with LUZ, a nonselective antagonist of MT, 24 h before the tests. As shown in the mRFP-GFP-LC3 adenovirus-transfected cells, the number of red puncta decreased in LUZ-treated cells compared to control cells, and the increase in red puncta mediated by MLT was also inhibited by LUZ treatment (Fig. 6a, b), suggesting that the restoration of autophagic flux was resuppressed by LUZ treatment. Consistently, LUZ reversed the MLT-induced expression changes in autophagy-related proteins, including Atg7, Beclin-1, LC3-II, and p62, which suggests that MLT restores autophagy in senescent cells in an MT-dependent manner (Fig. 6c, d). SA-β-gal staining (Fig. 6e) and Western blot analysis indicated that the expression of senescence-associated proteins, including p16, p21, p53, and γ-H2AX (Fig. 6f. g), and the senescence features of P15 cells stacked when LUZ was applied to block MT-dependent autophagy regardless of MLT treatment.

Next, we explored the possible downstream signaling pathway involved in receptor-dependent MLT-mediated autophagy regulation. As suggested in a previous study [29], MT is involved in the downregulation of PI3K/AKT by MLT, and the PI3K/AKT/mTOR pathway is recognized as a main signaling pathway of autophagy regulation. Hence, we assessed the expression of the phosphorylated forms of PI3K/AKT/mTOR in cells treated with LUZ. Western blotting indicated that the expression of p-PI3K, p-AKT, and p-mTOR in the LUZ-treated group was significantly increased compared to that in the control group, while LUZ had little effect on the total protein expression of PI3K, AKT, and mTOR. The results also showed that MLT treatment decreased the expression of p-PI3K, p-AKT, and p-mTOR but had no effect on the total protein expression (Fig. S4c). Moreover, the expression of both phosphorylated and total proteins of PI3K, AKT, and mTOR was comparable between the control group and the MLT and LUZ cotreatment group (Fig. S4c-e). In accordance with a former study [29], these results collectively suggest that MLT could negatively regulate the PI3K/AKT/mTOR pathway via MT.

### MLT restores autophagy and attenuates cellular senescence by downregulating the MT/PI3K/Akt/mTOR signaling pathway

**Fig. 7:**
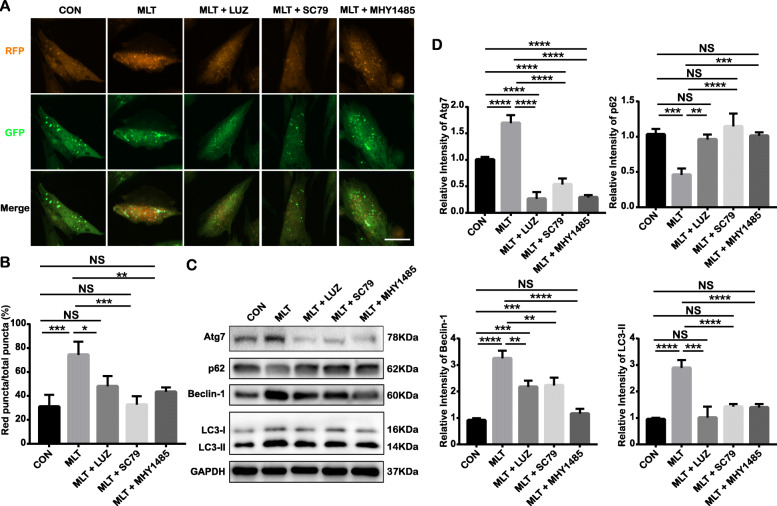
The MT/PI3K/AKT/mTOR pathway is involved in MLT-mediated autophagy regulation. (CON: P15 cells without any treatment; MLT: MLT-treated P15 cells; MLT + LUZ: MLT plus LUZ-treated P15 cells; MLT + SC79: MLT plus SC79-treated P15 cells; MLT + MHY1485: MLT plus MHY1485-treated P15 cells). *p < 0.05, **p < 0.01, ***p < 0.001, and ****p < 0.0001 represent significant differences between the indicated columns, while NS represents no significant difference. **a** Representative confocal images of autophagosomes (yellow) and their matured form, autolysosomes (red), in mRFP-GFP-LC3 adenovirus-transfected P15 cells with or without MLT/LUZ/SC79/MHY1485 treatment (scale bar = 50 μm, ×200 magnification). **b** Quantitative analysis of the percentage of red puncta out of the total puncta based on confocal examination. **c** Expression of cell autophagy-related proteins Atg7, p62, Beclin-1, and LC3-II in P15 cells with or without MLT/LUZ/SC79/MHY1485 treatment as detected by Western blot assay. **d** Quantitative analysis of Atg7, p62, Beclin-1, and LC3-II expression in P15 cells with or without MLT/LUZ/SC79/MHY1485 treatment

**Fig. 8:**
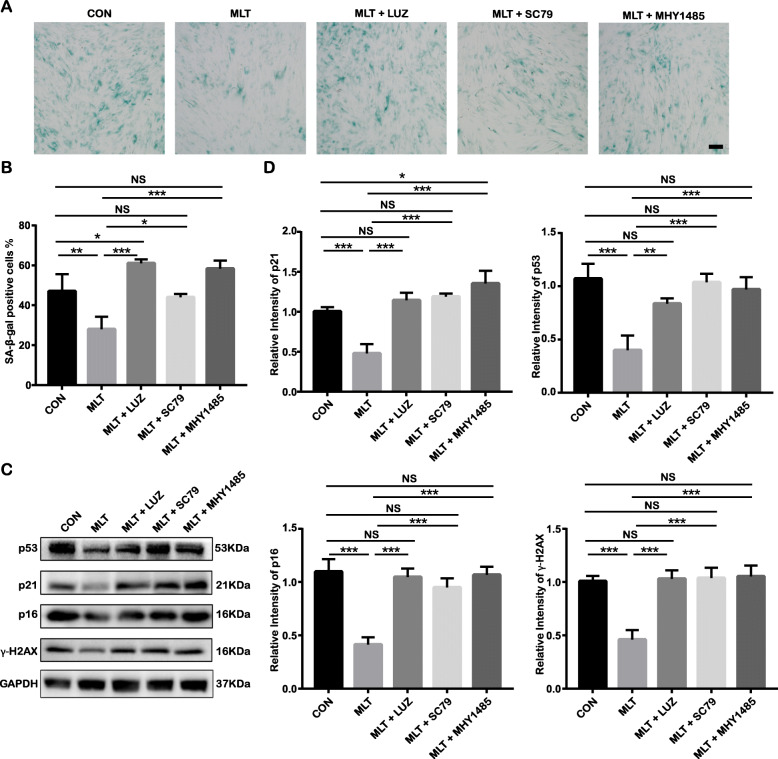
Downregulation of autophagy via MT/PI3K/AKT/mTOR signaling deteriorates senescence in cells undergoing long-term ex vivo passaging. (CON: P15 cells without any treatment; MLT: MLT-treated P15 cells; MLT + LUZ: MLT plus LUZ-treated P15 cells; MLT + SC79: MLT plus SC79-treated P15 cells; MLT + MHY1485: MLT plus MHY1485-treated P15 cells). *p < 0.05, **p < 0.01, ***p < 0.001, and ****p < 0.0001 represent significant differences between the indicated columns, while NS represents no significant difference. **a** Representative images of SA-β-gal expression in P15 cells with or without MLT/LUZ/SC79/MHY1485 treatment (immunocytochemical staining, positively stained cells were dyed in blue; scale bar = 100 μm). **b** Percentage of SA-β-gal-positive cells among all cells and quantitative analysis. **c** Cell senescence-related protein expression in P15 cells with or without MLT/LUZ/SC79/MHY1485 treatment as determined by Western blot assay. **d** Quantitative analysis of p53, p21, p16, and γ-H2AX expression in P15 cells with or without MLT/LUZ/SC79/MHY1485 treatment

To further verify the role of the MT/PI3K/Akt/mTOR pathway in MLT-regulated cellular autophagy and rejuvenation, we added the MT-specific blocker LUZ, the PI3K/Akt agonist SC79, and the mTOR selective agonist MHY1485 to MLT-treated cells. Compared to that in the MLT-treated group, autophagic flux was obstructed in the MLT + LUZ group, MLT + SC79 group, and MLT + MHY1485 group, as suggested by the decreased number of red puncta in the merged pictures of mRFP-GFP-LC3 adenovirus-transfected cells (Fig. 7a, b). Consistent results were also shown in terms of the expression of autophagy-associated proteins, including Atg7, Beclin-1, LC3-II, and p62, as evaluated by Western blotting (Fig. 7c, d). These results all suggested that MLT-induced autophagy in senescent cells was abolished by the MT blocker, PI3K/Akt agonist, and mTOR agonist, indicating the involvement of the MT/PI3K/Akt/mTOR axis in MLT-mediated autophagy restoration. These cotreatments (MLT + LUZ, MLT + SC79, and MLT + MHY1485) not only attenuated autophagy induction but also reversed the MLT-induced effects on senescence features, as evidenced by the increased number of SA-β-gal-positive cells (Fig. 8a, b), and the expression of senescence associated proteins including p16, p21, p53, and γ-H2AX (Fig. 8c, d). Collectively, these findings indicate that the downregulation of autophagy in senescent cells undergoing long-term ex vivo passaging occurs via the MT/PI3K/AKT/mTOR signaling pathways.

## Discussion

As an important cell source for therapeutic purposes, stem cells of dental origin, such as PDLSCs obtained from PDL tissue have gained increasing attention in recent years due to their MSC-like characteristics, including but not limited to multipotency, high proliferation rates, immunomodulatory properties, and tissue repair capacity. In this context, PDLSCs can be easily obtained from donor teeth extracted due to impaction (i.e., third molars) or orthodontic reasons with negligible ethical concerns, and definite evidence validates their value, as the first choice of MSCs, for the treatment of periodontal disease as well as therapeutic potential in multiple areas beyond the scope of the stomatognathic system. However, the amount of PDL tissue available for the isolation of PDLSCs is relatively limited; hence, it is usually not feasible to obtain a clinically relevant quantity of cells merely by isolation. To bridge the gap between the limited number of acquirable stem cells from donors and the large quantity of stem cells needed for therapeutic use, it is necessary to apply long-term in vitro passaging for large-scale cell production, especially for autologous MSC transplantation [7]. However, accumulating evidence suggests that long-term passaging might cause cellular senescence with unexpected alterations, including anomalous morphological changes [8], hindered proliferation capacity [34], and decreased differentiation potential in terms of chondrogenesis and osteogenesis [35]. In general, given that studies suggest that the stemness of MSCs is only be retained up to 6 passages and significantly declines after 10 passages, to avoid senescence-associated alterations, P2 to P5 cells, which are believed to possess self-renewal and differentiation capacities analogous to those of P0 cells, are often chosen as the objects of study [36]. Another study also suggested that long-term in vitro expansion significantly decreases the therapeutic properties, including proliferation, differentiation, and immunomodulation, of MSCs [26]. These compromised characteristics of MSCs could prominently decrease the therapeutic efficacy and effectiveness of cellular therapy for tissue repair and regeneration. Here, we chose P2 cells as the young group and P7 and P15 cells as the senescent groups to study senescence-associated alterations that occur along with long-term in vitro expansion. To ensure that comparisons were made among various passages of PDLSCs derived from the same donor, cryopreserved P2, P7, and P15 cells were used in the present study and thawed at the same time prior to investigation. Consistent with previous investigations, our study revealed that in PDLSCs, long-term in vitro expansion led to senescence, evidenced by decreases in proliferation and osteogenic differentiation and increases in SA-β-gal activity and the expression of senescence-associated proteins p16, p21, p53, and γ-H2AX, which together symbolize cell cycle arrest and DNA damage. Therefore, we verified in PDLSCs that long-term in vitro expansion caused cellular senescence, as was found in other kinds of MSCs, and indicated the necessity of rejuvenation during in vitro expansion.

To overcome the challenge of senescence during in vitro passaging, it is necessary to identify the mechanism underlying long-term expansion-caused senescence. It has been revealed that autophagy deficiency is concomitant with age-related disorders [32, 37], organismal aging [38], and the senescence of stem cells extracted from physically aging animal models [12, 13]. Furthermore, inhibition of autophagy can accelerate neurodegenerative disease by facilitating the accumulation of disease-causing aggregate-prone proteins [39] and inducing kidney damage in aging mice [40]. These results indicate the interconnection between autophagy and senescence. To our knowledge, the autophagy level of senescent stem cells after long-term passaging has not been studied in detail. Consistent with physiological or disease-related organismal and cellular senescence, a decline in basal autophagy level was first found in long-term in vitro expansion-caused cellular senescence in our study; this phenomenon was evidenced by the inhibition of autophagic flux, the decreases of expression of autophagy-related proteins including Atg7, Beclin-1, and LC3-II and the accumulation of the autophagy substrate p62. To further verify the connection between autophagy alteration and cellular senescence, the autophagy inhibitor 3-MA was added to cells. We found that 3-MA-mediated autophagy inhibition could deteriorate cell senescence, as evidenced by elevated SA-β-gal activity, increased DNA damage, and cell cycle arrest. On the other hand, previous studies showed that autophagy restoration could alleviate senescence and achieve functional improvement. Studies suggested that treatment with urolithin A to induce autophagy attenuated the age-related decline in muscle function and enhanced exercise capacity in old rodents [41]; autophagy restoration mediated by caloric restriction could decrease the cellular ROS level and enhance the regenerative ability of aging stem cells [42]; stimulation of lysosomal activity to promote autophagy in aged neural stem cells significantly enhanced their neural regeneration ability [43]. These results collectively indicate that the regulation of autophagy could be a potential strategy for the functional maintenance of senescent stem cells.

To date, researchers have made great efforts to restore the functionality of senescent stem cells. The strategies to reach optimum cultivation for restoring or promoting the capacity of stem cells can be generally divided into three categories. The first strategy is to mimic the in vivo microenvironment and restore the expression of cytoprotective and stemness-associated genes by providing an extracellular matrix (ECM)-simulated environment, by short-term hypoxia preconditioning and by other means. The second strategy is to utilize exogenous molecules to activate extracellular or intracellular signaling to resist the harsh in situ microenvironment caused by ischemia, nutrition deprivation, and oxidative damage by pretreating cells with exogenous cytokines, growth factors and other small molecule drugs. The third strategy involves the genetic modulation of stem cells to facilitate the acquisition of superior capacities in self-protection, proliferation, and differentiation. Although progress has been made using former strategies, additional problems have arisen. Hypoxic preconditioning is reported to induce cell cycle arrest and inhibit cell proliferation [44, 45]. The utilization of exogenous signaling proteins seems to have relatively low efficiency and high cost because of their short half-lives [46]. Genetic engineering may have a risk of insertional mutagenesis and oncogene transaction. Thus, other strategies with both high efficiency and safe applications are needed to restore the functionality of senescent stem cells. As confirmed in recent studies, MLT exhibits unique superiority in tissue engineering. As a pleiotropic endogenous hormone secreted by the pineal gland and almost every other tissue, MLT exhibits low toxicity in both animal and clinical studies [47, 48]. In several studies, MLT enhanced survival stability by attenuating apoptosis, protecting cells from oxidative stress and inflammation-induced damage [49], improving the chondrogenesis and osteogenesis of MSCs [50], and maintaining the stemness of long-term passaged stem cells, resulting in preserved proliferation, osteogenic differentiation, and immunomodulation capacities [26]. In addition to its well-known role in stem cell-based therapy as a probable free radical scavenger with excellent anti-inflammatory and differentiation enhancement properties, MLT was also recently found to function as a regulator of autophagy. In some studies, MLT increased the basal levels of autophagy under physiological conditions, maintained neuronal homeostasis and survival from a subarachnoid hemorrhage followed by brain injury [51], and modulated autophagy to attenuate cardiac ischemia/reperfusion injury [52, 53]. MLT-mediated regulation of autophagy could also benefit some age-related diseases. MLT application in vivo ameliorated AD-induced cardiac atrophy [28] and attenuated the decline in cognitive function in tau-related AD rats [27] by restoring autophagic flux. Moreover, MLT could attenuate the degradation of damaged mitochondria by mitophagy during aging and under neurodegenerative conditions [54]. Similar to the studies mentioned above, our study also confirmed that MLT could effectively restore cellular autophagy not only by elevating the basal autophagy level in senescent P15 cells but also by enhancing the lysosome-dependent degradation of autophagosomes. Our study also found that restored autophagy was further accompanied by decreased senescence. To further clarify whether restored autophagy was the reason for the rejuvenation effects, we applied the autophagy inhibitor 3-MA. We found that defective autophagy caused by 3-MA abrogated MLT-mediated rejuvenation. Therefore, our results suggest that autophagy restoration alleviated cellular senescence and that the manipulation of autophagy could be an effective strategy for maintaining the functionality of senescent cells.

To investigate the underlying mechanism of MLT-mediated autophagy restoration, we first explored whether the effect was dependent on MT. After affirming that PDLSCs expressed MT, we supplemented cultured cells with the MT-specific inhibitor LUZ. We found that LUZ further exacerbated autophagy inhibition in senescent cells without MLT treatment, enhancing cellular senescence. Even in senescent cells treated with MLT, LUZ abrogated the induction of autophagy mediated by MLT and further inhibited MLT-mediated cell rejuvenation. These results indicate that MLT-mediated autophagy restoration occurs in an MT-dependent manner in senescent cells. Moreover, mTOR plays an important role as a central switch in autophagy, while the downregulation of PI3K/AKT/mTOR pathway can activate autophagy. In these regards, several studies have targeted the PI3K/AKT/mTOR pathway for autophagy regulation in stem cells [55–57]. Accordingly, our study demonstrated that the expression of p-PI3K, p-AKT, and p-mTOR significantly decreased after MLT treatment but was restored with LUZ treatment. We thus concluded that MT-dependent MLT-mediated autophagy may occur through inhibition of the PI3K/AKT/mTOR pathway.

To precisely confirm that MLT-mediated autophagy occurs through the MT/PI3K/AKT/mTOR pathway, we cotreated cells with MLT and LUZ, the PI3K/AKT-specific agonist SC79, and the mTOR agonist MHY1485. We found that the MLT-mediated autophagy restoration and senescence attenuation could be abrogated by blocking MT and activating the PI3K/AKT/mTOR pathway. However, our study did not determine the exact type of MT involved in MLT-mediated autophagy restoration, which should be our main pursuit in the future.

In summary, our study revealed for the first time that autophagy inhibition is one of the vital mechanisms of cellular senescence induced by long-term ex vivo expansion and that autophagy can be restored by MLT through the MT/PI3K/AKT/mTOR pathway. These findings suggest that autophagy promoters, including but not limited to MLT, may be therapeutic agents in cellular therapy in the future, and reveal that MLT can be applied to restore autophagy to rejuvenate stem cells during long-term passaging. The investigation focused on the mechanism by PI3K/AKT/mTOR pathway inhibition could enhance autophagy to attenuate cellular senescence, which may provide a possible target for research and even clinical applications aimed at the rejuvenation of stem cells for cellular therapy.

## Conclusions

Large-scale production of cellular materials for use in a clinical setting necessitates safe and cost-effective agents that can be used to ensure the function and therapeutic properties of stem cells following long-term ex vivo expansion. Although the use of MLT as a cell-medium adjuvant has been proposed to prevent cells from senescence following long-term passaging, the underlying mechanism remains largely unexplored. In this study, we proved for the first time that the impaired autophagy of PDLSCs was a significant mechanism underlying cellular senescence caused by long-term in vitro expansion and that the function of MLT in combating cell aging was related to its role as an autophagy regulator. These findings suggest that autophagy promoters, including but not limited to MLT, may be used as therapeutic agents in cellular therapy in the future. Further investigations indicated that MT-dependent PI3K/AKT/mTOR signaling was involved in MLT-induced alterations in cell autophagy. This is the first report to demonstrate that MLT-induced cell rejuvenation occurs via the regulation and restoration of damaged cell autophagic processes, indicating that autophagy-restoring agents can be expoited to facilitate the development of optimized clinical-scale cell production protocols and hence to yield adequate cellular materials for cellular therapy and regenerative medicine.

## Supplementary Information

**Additional file 1: Supplementary Fig. 1.** Isolation and identification of human PDLSCs. Human PDLSCs were cultured and passaged in vitro; across the in vivo expansion stage, passage 2 (P2), passage 7 (P7), and passage 15 (P15) cells were collected for the following examinations. (**a**) Expression of cell surface markers in P2, P7 and P15 cells as determined by flow cytometry. (**b**) Chondrogenic differentiation in P2, P7 and P15 cells according to Alcian blue staining (representative images, scale bar = 100 μm). (**c**) Adipogenic differentiation in P2, P7 and P15 cells according to Oil Red O staining (representative images of lipid droplets, scale bar = 100 μm). (**d**) Osteogenic differentiation potential of P2, P7 and P15 cells as indicated by Alizarin Red staining (top: representative general views; bottom: representative images of calcium nodules, scale bar = 100 μm). (**e**) Quantitative analysis of Alizarin Red staining for P2, P7 and P15 cells (* *p* < 0.05 and ** *p* < 0.01 represent significant differences between the indicated columns). (**f**) ALP staining of P2, P7 and P15 cells (top: representative general views; bottom: representative images of ALP staining, scale bar = 100 μm). (**g**) Quantitative analysis of ALP activity in P2, P7 and P15 cells (** *p* < 0.01 and **** *p* < 0.0001 represent significant differences between the indicated columns).

**Additional file 2: Supplementary Fig. 2.** The selection of the optimal MLT concentration. * *p* < 0.05, ** *p* < 0.01, *** *p* < 0.001 and **** *p* < 0.0001 represent significant differences between the indicated columns, while NS represents no significant difference. (**a**) Cell viability of P15 cells, as evaluated by CCK-8 analysis following MLT treatment at the indicated concentrations. (**b**) Protein levels of autophagy-related proteins Atg7, p62, Beclin-1 and LC3 in P15 cells following MLT treatment at various concentrations (Western blot assay). (**c**) Quantitative analysis of Atg7, p62, Beclin-1 and LC3 expression in P15 cells following MLT treatment at the indicated concentrations. (**d**) Gene expression levels of cell senescence-related proteins (p53, p21, p16 and γ-H2AX) in P15 cells following MLT treatment at the indicated concentrations (qRT-PCR).

**Additional file 3: Supplementary Fig. 3.** MLT treatment promoted the proliferation and enhanced the osteogenic potential of P15 cells. * *p* < 0.05, ** *p* < 0.01, *** *p* < 0.001 and **** *p* < 0.0001 represent significant differences between the indicated columns, while NS represents no significant difference. (**a**) Proliferative activity of P2, P15 and P15 + MLT cells as determined by the CCK-8 assay. (**b**) Osteogenic differentiation potential of P2, P15 and P15+ MLT cells as determined by Alizarin Red staining (top: representative general views; bottom: representative images of calcium nodules, scale bar = 100 μm). (**c**) Quantitative analysis of Alizarin Red staining for P2, P15 and P15 + MLT cells (* *p* < 0.05 and ** *p* < 0.01 represent significant differences between the indicated columns). (**d**) ALP staining of P2, P15 and P15 + MLT (top: representative general views; bottom: representative images of ALP staining, scale bar = 100 μm). (**e**) Quantitative analysis of ALP activity in P2, P7 and P15 cells. (**f**) Osteoblast differentiation-related protein expression in P2, P15 and P15+ MLT cells as determined by Western blot assay. Quantitative analysis of (**g**) RUNX2 and (**h**) OCN expression in P2, P15 and P15+ MLT cells.

**Additional file 4: Supplementary Fig. 4.** MT is involved in the regulation of PI3K/AKT/mTOR signaling. (CON: P15 cells without any treatment; MLT: MLT-treated P15 cells; LUZ: LUZ-treated P15 cells; MLT + LUZ: MLT plus LUZ–treated P15 cells**).** * *p* < 0.05, ** *p* < 0.01, *** *p* < 0.001 and **** *p* < 0.0001 represent significant differences between the indicated columns, while NS represents no significant difference. (**a**) Melatonin receptor (MT) expression in P2, P7 and P15 cells was assessed by Western blot assay. (**b**) Quantitative analysis of MT expression in P2, P7 and P15 cells. (**c**) Protein levels of p-PI3K (Tyr458), p-AKT (Ser473), p-mTOR (Ser2448), PI3K, AKT and mTOR in P15 cells with or without MLT/LUZ treatment as determined by Western blot assay. (**d**) Quantitative analysis of the ratio of p-mTOR/mTOR in P15 cells with or without MLT/LUZ treatment. (**e**) Quantitative analysis of the ratio of p-PI3K/PI3K in P15 cells with or without MLT/LUZ treatment. (**f**) Quantitative analysis of the ratio of p-AKT/AKT in P15 cells with or without MLT/LUZ treatment.

## Data Availability

All data generated or analyzed during this study are included in this published article.

## Abbreviations

MSCs: Mesenchymal stromal cells
SA-β-gal: Senescence-associated β-galactosidase
ROS: Reactive oxygen species
MLT: Melatonin
AD: Alzheimer’s disease
MT: MLT receptor
PDL: Periodontal ligament
PDLSCs: Periodontal ligament stem cells
CCK-8: Cell Counting Kit-8
ALP: Alkaline phosphatase
3-MA: 3-Methyladenine
LUZ: Luzindole
Baf: Bafilomycin A1

## References

[1] Furlani D, Li W, Pittermann E, Klopsch C, Wang L, Knopp A, Jungebluth P, Thedinga E, Havenstein C, Westien I, Ugurlucan M, Li RK, Ma N, Steinhoff G (2009). A transformed cell population derived from cultured mesenchymal stem cells has no functional effect after transplantation into the injured heart. Cell Transplant.

[2] Bustos ML, Huleihel L, Kapetanaki MG, Lino-Cardenas CL, Mroz L, Ellis BM, McVerry BJ, Richards TJ, Kaminski N, Cerdenes N, Mora AL, Rojas M (2014). Aging mesenchymal stem cells fail to protect because of impaired migration and antiinflammatory response. Am J Respir Crit Care Med.

[3] Castorina A, Szychlinska M, Marzagalli R, Musumeci G (2015). Mesenchymal stem cells-based therapy as a potential treatment in neurodegenerative disorders: is the escape from senescence an answer?. Neural Regen Res.

[4] Sepúlveda JC, Tomé M, Eugenia Fernández M, Delgado M, Campisi J, Bernad A (2014). Cell senescence abrogates the therapeutic potential of human mesenchymal stem cells in the lethal endotoxemia model. Stem Cells.

[5] Davis DR, Stewart DJ (2011). Autologous cell therapy for cardiac repair. Expert Opin Biol Ther.

[6] Rigato M, Monami M, Fadini GP (2017). Autologous cell therapy for peripheral arterial disease: systematic review and meta-analysis of randomized, nonrandomized, and noncontrolled studies. Circ Res.

[7] Toma C, Wagner WR, Bowry S, Schwartz A, Villanueva F (2009). Fate of culture-expanded mesenchymal stem cells in the microvasculature: *in vivo* observations of cell kinetics. Circ Res.

[8] Korolchuk VI, Miwa S, Carroll B, von Zglinicki T (2017). Mitochondria in cell senescence: is mitophagy the weakest link?. EBioMedicine..

[9] Ren R, Ocampo A, Liu GH, Izpisua Belmonte JC (2017). Regulation of stem cell aging by metabolism and epigenetics. Cell Metab.

[10] Bork S, Pfister S, Witt H, Horn P, Korn B, Ho AD, Wagner W (2010). DNA methylation pattern changes upon long-term culture and aging of human mesenchymal stromal cells. Aging Cell.

[11] Jiang T, Xu G, Wang Q, Yang L, Zheng L, Zhao J, Zhang X (2017). *In vitro* expansion impaired the stemness of early passage mesenchymal stem cells for treatment of cartilage defects. Cell Death Dis.

[12] García-Prat L, Martínez-Vicente M, Perdiguero E, Ortet L, Rodríguez-Ubreva J, Rebollo E, Ruiz-Bonilla V, Gutarra S, Ballestar E, Serrano AL, Sandri M, Muñoz-Cánoves P (2016). Autophagy maintains stemness by preventing senescence. Nature..

[13] Revuelta M, Matheu A (2017). Autophagy in stem cell aging. Aging Cell.

[14] Ho TT, Warr MR, Adelman ER, Lansinger OM, Flach J, Verovskaya EV, Figueroa ME, Passegué E (2017). Autophagy maintains the metabolism and function of young and old stem cells. Nature..

[15] Acuña-Castroviejo D, Escames G, Venegas C, Díaz-Casado ME, Lima-Cabello E, López LC, Rosales-Corral S, Tan DX, Reiter RJ (2014). Extrapineal melatonin: sources, regulation, and potential functions. Cell Mol Life Sci.

[16] Reiter RJ (1991). Pineal melatonin: cell biology of its synthesis and of its physiological interactions. Endocr Rev.

[17] Reiter RJ, Tan DX, Galano A (2014). Melatonin: exceeding expectations. Physiology..

[18] Galano A, Tan D-X, Reiter R (2018). Melatonin: a versatile protector against oxidative DNA damage. Molecules..

[19] Bermejo-Millo JC, Guimarães MRM, de Luxán-Delgado B, Potes Y, Pérez-Martínez Z, Díaz-Luis A, Caballero B, Solano JJ, Vega-Naredo I, Coto-Montes A (2018). High-fructose consumption impairs the redox system and protein quality control in the brain of Syrian hamsters: therapeutic effects of melatonin. Mol Neurobiol.

[20] Luchetti F, Canonico B, Bartolini D, Arcangeletti M, Ciffolilli S, Murdolo G, Piroddi M, Papa S, Reiter RJ, Galli F (2014). Melatonin regulates mesenchymal stem cell differentiation: a review. J Pineal Res.

[21] Wu Z, Qiu X, Gao B, Lian C, Peng Y, Liang A, Xu C, Gao W, Zhang L, Su P, Rong L, Huang D (2018). Melatonin-mediated miR-526b-3p and miR-590-5p upregulation promotes chondrogenic differentiation of human mesenchymal stem cells. J Pineal Res.

[22] Zhou L, Chen X, Liu T, Gong Y, Chen S, Pan G, Cui W, Luo ZP, Pei M, Yang H, He F (2015). Melatonin reverses H2O2 -induced premature senescence in mesenchymal stem cells via the SIRT1-dependent pathway. J Pineal Res.

[23] Shuai Y, Liao L, Su X, Yu Y, Shao B, Jing H, Zhang X, Deng Z, Jin Y (2016). Melatonin treatment improves mesenchymal stem cells therapy by preserving stemness during long-term *in vitro* expansion. Theranostics..

[24] Pierpaoli W, Dall'Ara A, Pedrinis E, Regelson W (1991). The pineal control of aging. The effects of melatonin and pineal grafting on the survival of older mice. Ann N Y Acad Sci.

[25] Thomas JN, Smith-Sonneborn J (1997). Supplemental melatonin increases clonal lifespan in the protozoan *Paramecium tetraurelia*. J Pineal Res.

[26] Kędziora-Kornatowska K, Szewczyk-Golec K, Czuczejko J, Lumen KVMD, Pawluk H, Motyl J (2007). Effect of melatonin on the oxidative stress in erythrocytes of healthy young and elderly subjects. J Pineal Res.

[27] Luengo E, Buendia I, Fernández-Mendívil C, Trigo-Alonso P, Negredo P, Michalska P (2019). Pharmacological doses of melatonin impede cognitive decline in tau-related Alzheimer models, once tauopathy is initiated, by restoring the autophagic flux. J Pineal Res.

[28] Wang S, Wang L, Qin X, Turdi S, Sun D, Culver B, Reiter RJ, Wang X, Zhou H, Ren J (2020). ALDH2 contributes to melatonin-induced protection against APP/PS1 mutation-prompted cardiac anomalies through cGAS-STING-TBK1-mediated regulation of mitophagy. Signal Transduct Target Ther.

[29] Li Z, Li X, Chen C, Chan MTV, Wu WKK, Shen J (2017). Melatonin inhibits nucleus pulposus (NP) cell proliferation and extracellular matrix (ECM) remodeling via the melatonin membrane receptors mediated PI3K-Akt pathway. J Pineal Res.

[30] Janku F, McConkey DJ, Hong DS, Kurzrock R (2011). Autophagy as a target for anticancer therapy. Nat Rev Clin Oncol.

[31] Wallroth A, Koch PA, Marat AL, Krause E, Haucke V (2019). Protein kinase N controls a lysosomal lipid switch to facilitate nutrient signalling via mTORC1. Nat Cell Biol.

[32] Dikic I, Elazar Z (2018). Mechanism and medical implications of mammalian autophagy. Nat Rev Mol Cell Biol..

[33] Kabeya Y, Mizushima N, Ueno T, Yamamoto A, Kirisako T, Noda T, Kominami E, Ohsumi Y, Yoshimori T (2000). LC3, a mammalian homologue of yeast Apg8p, is localized in autophagosome membranes after processing. EMBO J.

[34] Stolzing A, Jones E, McGonagle D, Scutt A (2008). Age-related changes in human bone marrow-derived mesenchymal stem cells: consequences for cell therapies. Mech Ageing Dev.

[35] Shen C, Jiang T, Zhu B, Le Y, Liu J, Qin Z (2018). *In vitro* culture expansion impairs chondrogenic differentiation and the therapeutic effect of mesenchymal stem cells by regulating the unfolded protein response. J Biol Eng.

[36] Gharibi B, Hughes FJ (2012). Effects of medium supplements on proliferation, differentiation potential, and *in vitro* expansion of mesenchymal stem cells. Stem Cells Transl Med.

[37] Levine B, Kroemer G (2008). Autophagy in the pathogenesis of disease. Cell..

[38] Hansen M, Rubinsztein DC, Walker DW (2018). Autophagy as a promoter of longevity: insights from model organisms. Nat Rev Mol Cell Biol.

[39] Cullup T, Kho AL, Dionisi-Vici C, Brandmeier B, Smith F, Urry Z, Simpson MA, Yau S, Bertini E, McClelland V, al-Owain M, Koelker S, Koerner C, Hoffmann GF, Wijburg FA, Hoedt AE, Rogers RC, Manchester D, Miyata R, Hayashi M, Said E, Soler D, Kroisel PM, Windpassinger C, Filloux FM, al-Kaabi S, Hertecant J, del Campo M, Buk S, Bodi I, Goebel HH, Sewry CA, Abbs S, Mohammed S, Josifova D, Gautel M, Jungbluth H (2013). Recessive mutations in EPG5 cause Vici syndrome, a multisystem disorder with defective autophagy. Nat Genet.

[40] Yamamoto T, Takabatake Y, Kimura T, Takahashi A, Namba T, Matsuda J, Minami S, Kaimori JY, Matsui I, Kitamura H, Matsusaka T, Niimura F, Yanagita M, Isaka Y, Rakugi H (2016). Time-dependent dysregulation of autophagy: implications in aging and mitochondrial homeostasis in the kidney proximal tubule. Autophagy..

[41] Ryu D, Mouchiroud L, Andreux PA, Katsyuba E, Moullan N, Nicolet-dit-Félix AA (2016). Urolithin A induces mitophagy and prolongs lifespan in C. elegans and increases muscle function in rodents. Na Med.

[42] Bi S, Wang H, Kuang W (2018). Stem cell rejuvenation and the role of autophagy in age retardation by caloric restriction: an update. Mech Ageing Dev.

[43] Audesse AJ, Webb AE (2018). Enhancing lysosomal activation restores neural stem cell function during aging. J Exp Neurosci.

[44] Eliasson P, Rehn M, Hammar P, Larsson P, Sirenko O, Flippin LA (2010). Hypoxia mediates low cell-cycle activity and increases the proportion of long-term–reconstituting hematopoietic stem cells during *in vitro* culture. Exp Hematol.

[45] Guitart AV, Hammoud M, Dello Sbarba P, Ivanovic Z, Praloran V (2010). Slow-cycling/quiescence balance of hematopoietic stem cells is related to physiological gradient of oxygen. Exp Hematol.

[46] Chen F-M, Shelton RM, Jin Y, Chapple ILC (2009). Localized delivery of growth factors for periodontal tissue regeneration: role, strategies, and perspectives. Med Res Rev.

[47] Jahnke G, Marr M, Myers C, Wilson R, Travlos G, Price C (1999). Maternal and developmental toxicity evaluation of melatonin administered orally to pregnant Sprague-Dawley rats. Toxicol Sci.

[48] Sanchez-Barcelo EJ, Mediavilla MD, Tan DX, Reiter RJ (2010). Clinical uses of melatonin: evaluation of human trials. Curr Med Chem.

[49] Lee MS, Yin TC, Sung PH, Chiang JY, Sun CK, Yip HK (2017). Melatonin enhances survival and preserves functional integrity of stem cells: a review. J Pineal Res.

[50] Gao B, Gao W, Wu Z, Zhou T, Qiu X, Wang X, Lian C, Peng Y, Liang A, Qiu J, Zhu Y, Xu C, Li Y, Su P, Huang D (2018). Melatonin rescued interleukin 1β-impaired chondrogenesis of human mesenchymal stem cells. Stem Cell Res Ther.

[51] Chen J, Wang L, Wu C, Hu Q, Gu C, Yan F, Li J, Yan W, Chen G (2014). Melatonin-enhanced autophagy protects against neural apoptosis via a mitochondrial pathway in early brain injury following a subarachnoid hemorrhage. J Pineal Res.

[52] Zhou H, Li D, Zhu P, Hu S, Hu N, Ma S, Zhang Y, Han T, Ren J, Cao F, Chen Y (2017). Melatonin suppresses platelet activation and function against cardiac ischemia/reperfusion injury via PPARγ/FUNDC1/mitophagy pathways. J Pineal Res.

[53] Zhang Y, Wang Y, Xu J, Tian F, Hu S, Chen Y, Fu Z (2019). Melatonin attenuates myocardial ischemia-reperfusion injury via improving mitochondrial fusion/mitophagy and activating the AMPK-OPA1 signaling pathways. J Pineal Res.

[54] Fujikake N, Shin M, Shimizu S (2018). Association between autophagy and neurodegenerative diseases. Front Neurosci.

[55] Xu Y, Tan M, Ma X, Li H, He X, Chen Z (2020). Human mesenchymal stem cells–derived conditioned medium inhibits hypoxia-induced death of neonatal porcine islets by inducing autophagy. Xenotransplantation..

[56] Wang M, Liang X, Cheng M, Yang L, Liu H, Wang X, Sai N, Zhang X (2019). Homocysteine enhances neural stem cell autophagy in *in vivo* and *in vitro* model of ischemic stroke. Cell Death Dis.

[57] Wu Z, Lu H, Yao J, Zhang X, Huang Y, Ma S, Zou K, Wei Y, Yang Z, Li J, Zhao J (2019). GABARAP promotes bone marrow mesenchymal stem cells-based the osteoarthritis cartilage regeneration through the inhibition of PI3K/AKT/mTOR signaling pathway. J Cell Physiol.

