# Supplementary figures and images for: Melatonin induces the rejuvenation of long-term ex vivo expanded periodontal ligament stem cells by modulating the autophagic process
Source: Stem Cell Res Ther. 2021 Apr 29;12:254. doi: 10.1186/s13287-021-02322-9 (PMC8082824; doi:10.1186/s13287-021-02322-9)

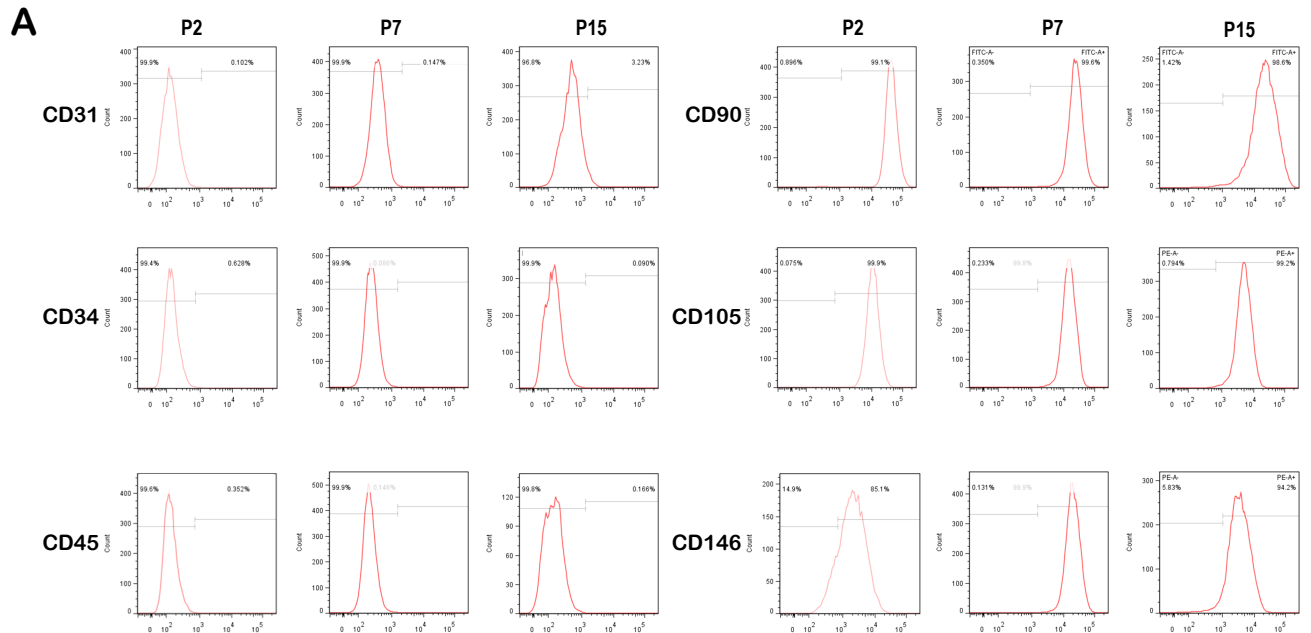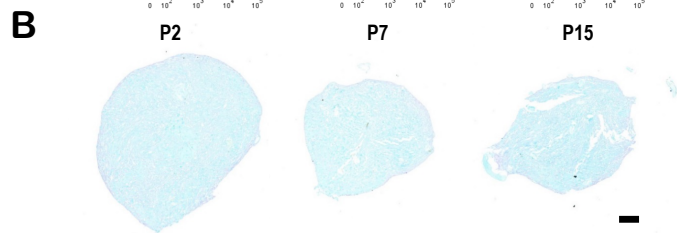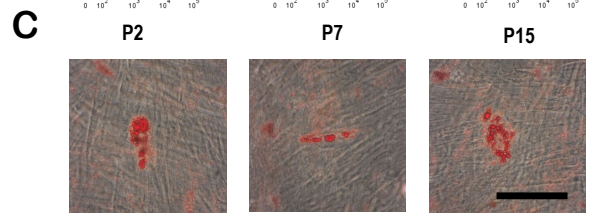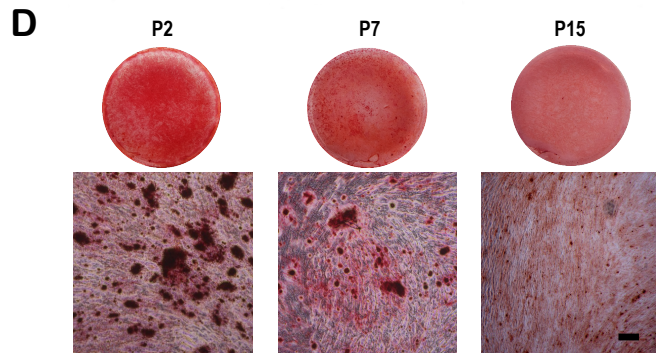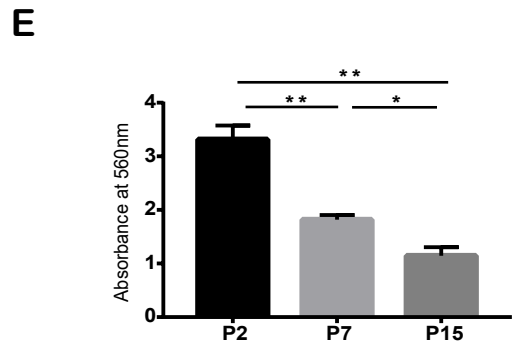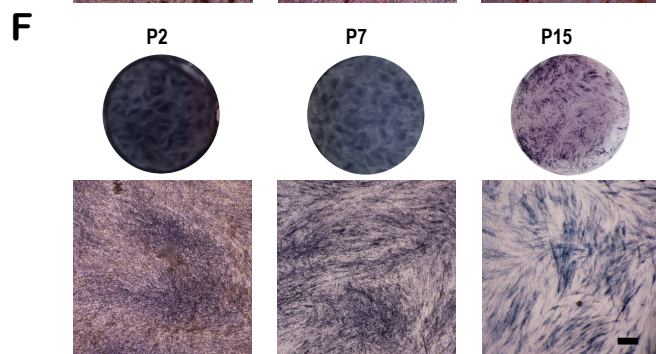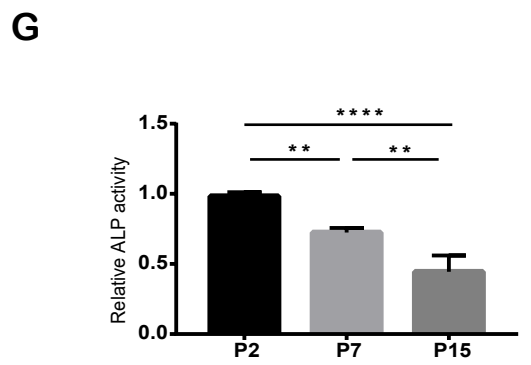

Supplement: Supplementary file 1 — Additional file 1: Supplementary Fig. 1. Isolation and identification of human PDLSCs. Human PDLSCs were cultured and passaged in vitro; across the in vivo expansion stage, passage 2 (P2), passage 7 (P7), and passage 15 (P15) cells were collected for the following examinations. (a) Expression of cell surface markers in P2, P7 and P15 cells as determined by flow cytometry. (b) Chondrogenic differentiation in P2, P7 and P15 cells according to Alcian blue staining (representative images, scale bar = 100 μm). (c) Adipogenic differentiation in P2, P7 and P15 cells according to Oil Red O staining (representative images of lipid droplets, scale bar = 100 μm). (d) Osteogenic differentiation potential of P2, P7 and P15 cells as indicated by Alizarin Red staining (top: representative general views; bottom: representative images of calcium nodules, scale bar = 100 μm). (e) Quantitative analysis of Alizarin Red staining for P2, P7 and P15 cells (* p < 0.05 and ** p < 0.01 represent significant differences between the indicated columns). (f) ALP staining of P2, P7 and P15 cells (top: representative general views; bottom: representative images of ALP staining, scale bar = 100 μm). (g) Quantitative analysis of ALP activity in P2, P7 and P15 cells (** p < 0.01 and **** p < 0.0001 represent significant differences between the indicated columns). [file 13287_2021_2322_MOESM1_ESM.pdf]

**A**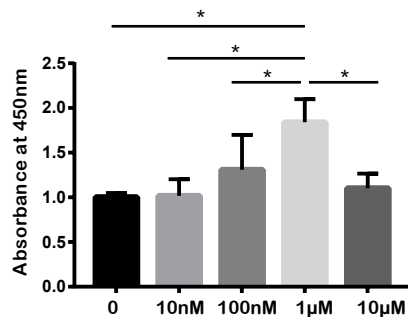**B**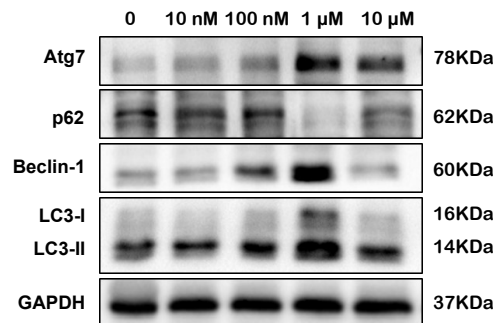**C**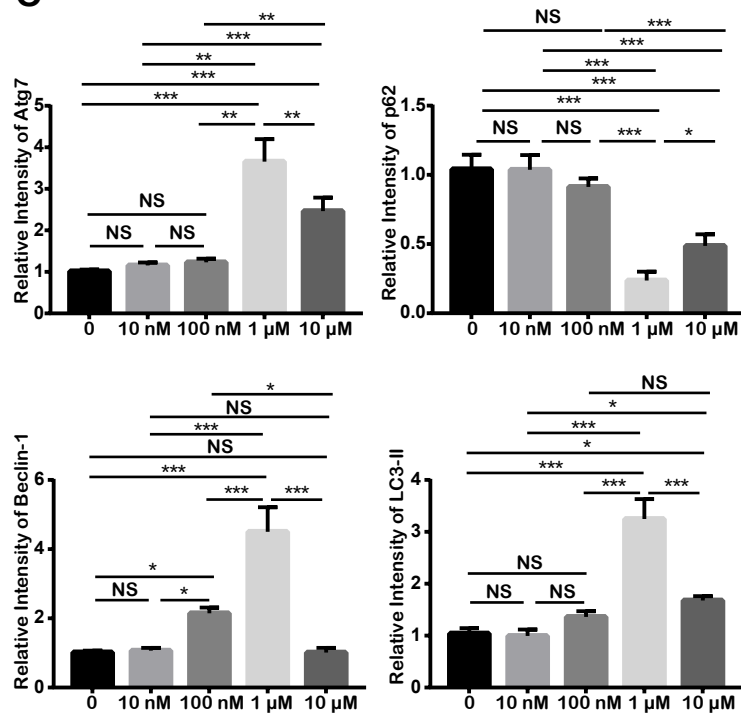**D**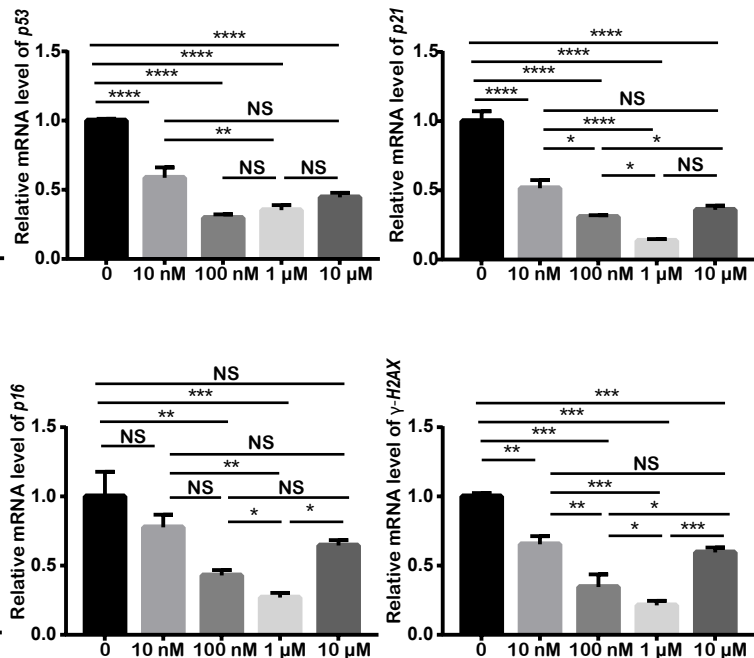

Supplement: Supplementary file 2 — Additional file 2: Supplementary Fig. 2. The selection of the optimal MLT concentration. * p < 0.05, ** p < 0.01, *** p < 0.001 and **** p < 0.0001 represent significant differences between the indicated columns, while NS represents no significant difference. (a) Cell viability of P15 cells, as evaluated by CCK-8 analysis following MLT treatment at the indicated concentrations. (b) Protein levels of autophagy-related proteins Atg7, p62, Beclin-1 and LC3 in P15 cells following MLT treatment at various concentrations (Western blot assay). (c) Quantitative analysis of Atg7, p62, Beclin-1 and LC3 expression in P15 cells following MLT treatment at the indicated concentrations. (d) Gene expression levels of cell senescence-related proteins (p53, p21, p16 and γ-H2AX) in P15 cells following MLT treatment at the indicated concentrations (qRT-PCR). [file 13287_2021_2322_MOESM2_ESM.pdf]

**A**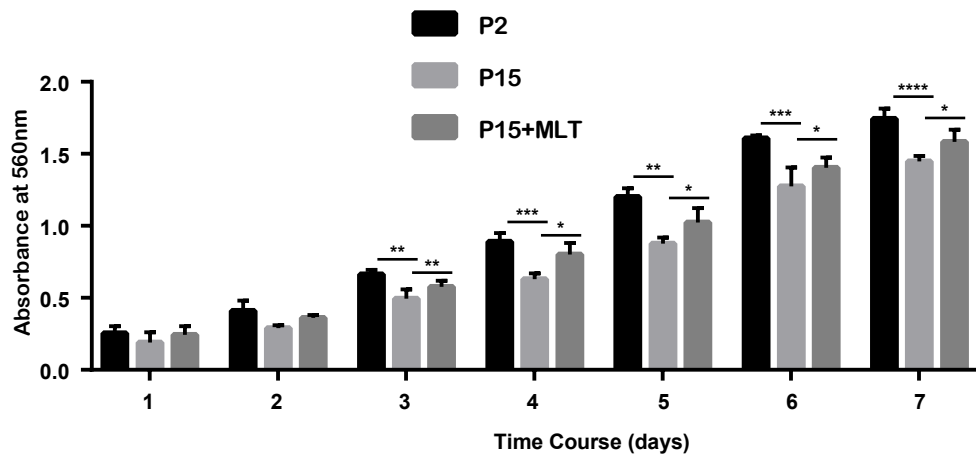**B**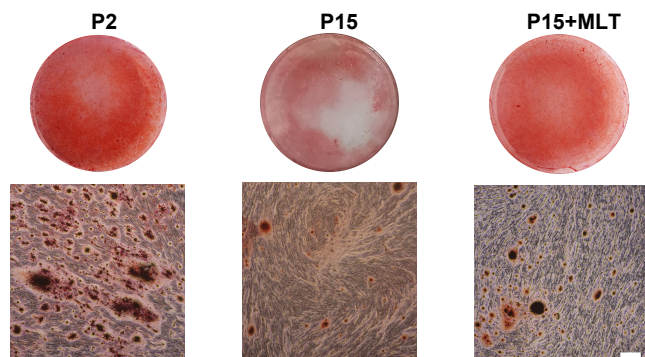**C**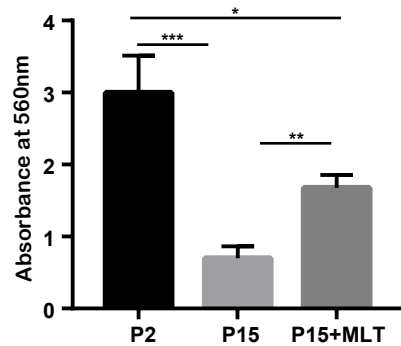**D**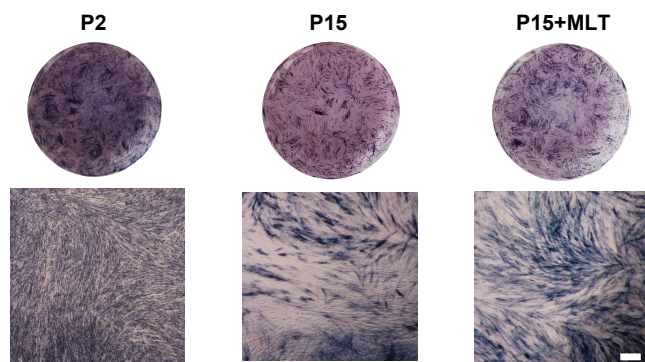**E**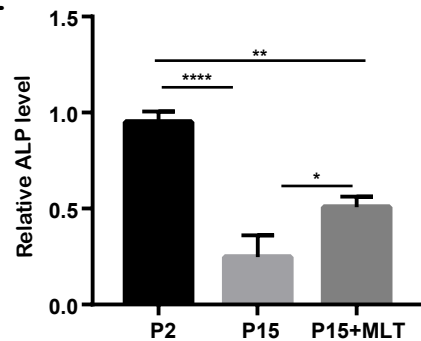**F**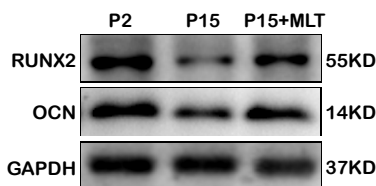**G**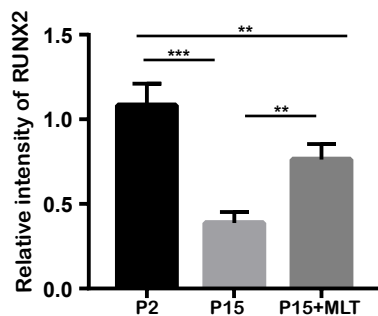**H**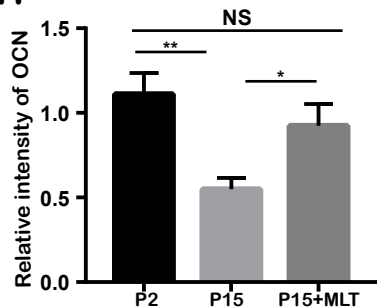

Supplement: Supplementary file 3 — Additional file 3: Supplementary Fig. 3. MLT treatment promoted the proliferation and enhanced the osteogenic potential of P15 cells. * p < 0.05, ** p < 0.01, *** p < 0.001 and **** p < 0.0001 represent significant differences between the indicated columns, while NS represents no significant difference. (a) Proliferative activity of P2, P15 and P15 + MLT cells as determined by the CCK-8 assay. (b) Osteogenic differentiation potential of P2, P15 and P15+ MLT cells as determined by Alizarin Red staining (top: representative general views; bottom: representative images of calcium nodules, scale bar = 100 μm). (c) Quantitative analysis of Alizarin Red staining for P2, P15 and P15 + MLT cells (* p < 0.05 and ** p < 0.01 represent significant differences between the indicated columns). (d) ALP staining of P2, P15 and P15 + MLT (top: representative general views; bottom: representative images of ALP staining, scale bar = 100 μm). (e) Quantitative analysis of ALP activity in P2, P7 and P15 cells. (f) Osteoblast differentiation-related protein expression in P2, P15 and P15+ MLT cells as determined by Western blot assay. Quantitative analysis of (g) RUNX2 and (h) OCN expression in P2, P15 and P15+ MLT cells. [file 13287_2021_2322_MOESM3_ESM.pdf]

**A**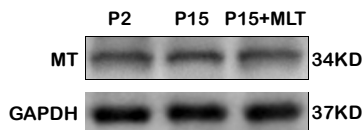**B**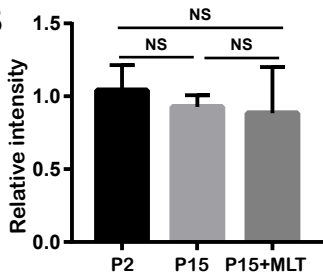**C**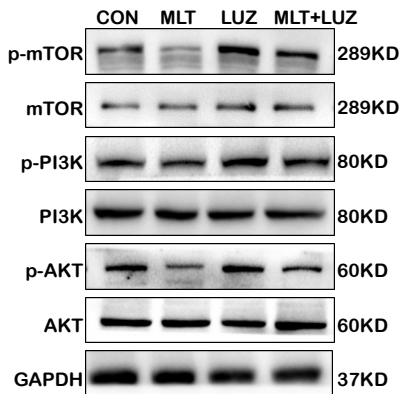**D**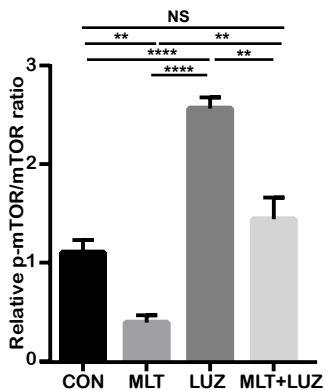**E**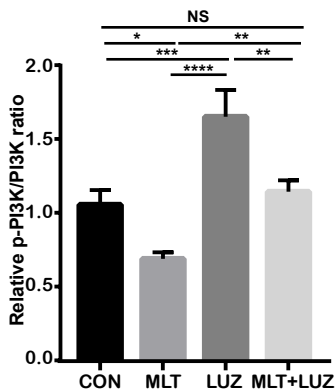**F**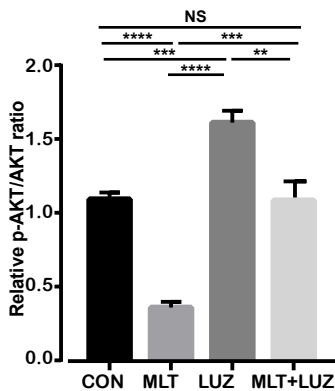

Supplement: Supplementary file 4 — Additional file 4: Supplementary Fig. 4. MT is involved in the regulation of PI3K/AKT/mTOR signaling. (CON: P15 cells without any treatment; MLT: MLT-treated P15 cells; LUZ: LUZ-treated P15 cells; MLT + LUZ: MLT plus LUZ–treated P15 cells). * p < 0.05, ** p < 0.01, *** p < 0.001 and **** p < 0.0001 represent significant differences between the indicated columns, while NS represents no significant difference. (a) Melatonin receptor (MT) expression in P2, P7 and P15 cells was assessed by Western blot assay. (b) Quantitative analysis of MT expression in P2, P7 and P15 cells. (c) Protein levels of p-PI3K (Tyr458), p-AKT (Ser473), p-mTOR (Ser2448), PI3K, AKT and mTOR in P15 cells with or without MLT/LUZ treatment as determined by Western blot assay. (d) Quantitative analysis of the ratio of p-mTOR/mTOR in P15 cells with or without MLT/LUZ treatment. (e) Quantitative analysis of the ratio of p-PI3K/PI3K in P15 cells with or without MLT/LUZ treatment. (f) Quantitative analysis of the ratio of p-AKT/AKT in P15 cells with or without MLT/LUZ treatment. [file 13287_2021_2322_MOESM4_ESM.pdf]
